# Superficially Similar Adaptation Within One Species Exhibits Similar Morphological Specialization but Different Physiological Regulations and Origins

**DOI:** 10.3389/fcell.2020.00300

**Published:** 2020-05-08

**Authors:** Yi Zhang, Xing-Xing Wang, Zhu-Jun Feng, Hao-Su Cong, Zhan-Sheng Chen, Yu-Dan Li, Wen-Meng Yang, Song-Qi Zhang, Ling-Feng Shen, Hong-Gang Tian, Yi Feng, Tong-Xian Liu

**Affiliations:** Key Laboratory of Integrated Pest Management on Crops in Northwestern Loess Plateau, Ministry of Agriculture, Northwest A&F University, Yangling, China

**Keywords:** environmental adaptation, *Harmonia axyridis*, melanization, multicolored Asian ladybird, phenotypic plasticity

## Abstract

Animals have developed numerous strategies to contend with environmental pressures. We observed that the same adaptation strategy may be used repeatedly by one species in response to a certain environmental challenge. The ladybird *Harmonia axyridis* displays thermal phenotypic plasticity at different developmental stages. It is unknown whether these superficially similar temperature-induced specializations share similar physiological mechanisms. We performed various experiments to clarify the differences and similarities between these processes. We examined changes in the numbers and sizes of melanic spots in pupae and adults, and confirmed similar patterns for both. The dopamine pathway controls pigmentation levels at both developmental stages of *H. axyridis.* However, the aspartate-β-alanine pathway controls spot size and number only in the pupae. An upstream regulation analysis revealed the roles of *Hox* genes and elytral veins in pupal and adult spot formation. Both the pupae and the adults exhibited similar morphological responses to temperatures. However, they occurred in different body parts and were regulated by different pathways. These phenotypic adaptations are indicative of an effective thermoregulatory system in *H. axyridis* and explains how insects contend with certain environmental pressure based on various control mechanisms.

## Introduction

Many animal species including insects modify their morphology to adapt to rapidly changing environmental conditions. Similar physiological and biological characteristics may be observed more than once within the same species. The multicolored Asian ladybird *Harmonia axyridis* (Pallas) is a ubiquitous insect pest predator that displays number of color patterns (Tan, 1946; Koch, 2003; Michie et al., 2010). The pupae present with only one gradually changeable melanic spot pattern, with an orange background and numerous dark spots. In contrast, the adults have discrete elytral patterns with background and spot color either orange or black. This plasticity is affected by both temperature and genetic background (Majerus et al., 2006; Michie et al., 2010, 2011; Knapp and Nedvěd, 2013). Recent studies established that the *pnr* gene determines elytra pattern background color in various color forms in *H. axyridis* (Ando et al., 2018; Gautier et al., 2018). However, the physiological and molecular mechanisms regulating thermally-induced spot size, shape, and number remain unclear.

The spot patterns of *H. axyridis* f. *succinea* are highly polymorphic across seasons and are temperature dependent. Seasonal phenotypic plasticity is advantageous for predictable environmental changes (Majerus et al., 2006; Michie et al., 2010, 2011; Knapp and Nedvěd, 2013). The adults and the pupae have low melanic body color (fewer and smaller spots) at high temperatures (Michie et al., 2010, 2011). This plasticity of body spot patterns to temperature is a thermal adaptation in *H. axyridis*. Dark-colored individuals rapidly increase their body temperature to warming by solar irradiation (Trullas et al., 2007). Similar thermal polyphenisms occur in butterflies and *Drosophila*. Cuticular melanization increases with decreasing temperature (Roland, 1982; Kingsolver, 1987; Gibert et al., 2000, 2016; Solensky and Larkin, 2003).

The numbers, sizes, and colors of the spots vary on the pupal dorsal cuticles and the adult elytra in *H. axyridis* f. *succinea* (Michie et al., 2011). Studies have shown that tyrosine-mediated cuticle pigmentation (melanization) plays a major role in cuticular melanin formation in numerous insect species (Michie et al., 2011; Sun et al., 2018a, b).

Insect cuticular melanization pathway is conserved among species. Tyrosine hydroxylase (TH) converts tyrosine to 3,4-dihydroxyphenylalanine (DOPA), and DOPA decarboxylase (DDC) converts DOPA to dopamine. The latter is a substrate for *N*-acetyldopamine (NADA) and *N*-β-alanyldopamine (NBAD) synthesized by arylalkylamine-*N*-acetyltransferase and NBAD synthase (ebony), respectively. *N*-acetyldopamine and NBAD are transported extracellularly and catalyzed by laccases (laccase 2, lac2) in cuticular melanization (pigmentation) and sclerotization (Suderman et al., 2006; Noh et al., 2016). Dopachrome is catalyzed by enzyme yellow to 5,6-dihydroxyindole-2-carboxylic acid, which polymerizes DOPA melanin. Aspartate decarboxylase (ADC) converts aspartic acid to β-alanine. The latter and dopamine are key substrates for ebony in NBAD formation. *ADC* and *ebony* downregulation limits β-alanine synthesis for NBAD production, leads to the accumulation of the melanin substrate dopamine, and enhances melanization (Borycz et al., 2002; Dai et al., 2015; Miyagi et al., 2015). Previous studies have shown that ebony and yellow determine spot patterns in numerous insect species (Wittkopp et al., 2002; Parchem et al., 2007; Futahashi et al., 2008, 2010; Wittkopp and Beldade, 2009; Arakane et al., 2010; Sharma et al., 2016).

*Harmonia axyridis*, that selected to investigate in this experiment, has been deployed as a biological control agent worldwide (Coderre et al., 1995; Dreistadt et al., 1995; Brown et al., 2007). The potential ecological impacts of introduction of *H. axyridis* must be considered (Koch, 2003; Koch and Galvan, 2007). Its environmental adaptation mediated by thermal phenotypic plasticity could be one reason that accounts for its global dispersal and possible negative ecological impact.

Thermal phenotypic plasticity is a major factor contributing to *H. axyridis* polymorphism. It is highly diverse and is directly induced by environmental stimuli. Melanic spot specialization is similar between pupae and adults. In this study, we examined the spot patterns in *H. axyridis* and their transcriptional regulation. The aims of this study were to compare thermally driven morphological changes at the phenotypical, physiological, and molecular levels in *H. axyridis* and to elucidate the mechanisms regulating its pigmentation patterns. Furthermore, we intended to compare the regulation differences of melanic spots formation between pupal dorsal cuticle and adult elytra to test our hypothesis that superficially similar phenotypic specification can be regulated through different molecular pathways.

## Materials and Methods

### Insects

Multicolored Asian ladybird (*H. axyridis*; orange background with dark spots; f. *succinea*) were maintained in a laboratory (Yangling, Shannxi, China) at 27.5 ± 1°C, 50% relative humidity (RH), and a photoperiod of 14-h light and 10-h day cycle for >2 years. The beetles were reared on pea aphids (*Acyrthosiphon pisum*) fed with broad bean (*Vicia faba*).

### Similar Morphological Change Patterns

#### Pupae

*H. axyridis* f. *succinea* larvae were reared at 15, 17.5, 20, 22.5, 25, 27.5, 30, 32.5, and 35°C (50% RH, 10,000 lx, 16L:8D) and prepared for sample collection and images analysis. Pupal samples were collected for both whole-body and segmental melanic spots analyses. Melanin spots of *H. axyridis* are color-uniform and have defined edges between them; therefore, the melanin levels can be calculated based on spots area proportion from images by pixels.

Top-, front-, and side-view images of the pupae ≥12 h post-pupation were captured with a Panasonic DMC-GH4 digital camera (Panasonic, Osaka, Japan; shutter speed: 1/250; aperture: F4.0; ISO: 320; picture style: faithful 0,0,0,0; white balance: color temperature, 3,000 K, AF mode: manual focus; metering mode: center-weighted average) coupled to the SDPTOP-SZN71 microscope system (Sunny, Hangzhou, Zhejiang, China; halogen lamp light temperature: 3,000 K). The percentages of spots on the whole body were calculated in ImageJ (v. 1.51j8; Wayne Rasband; National Institute of Health, Bethesda, MD, United States) and based on the spot area pixels proportions of whole-body pixels (projected areas, Supplementary Figure S3C). The melanin levels in all segments were also calculated (based on the spot area pixels proportions of all pixels for each segments).

First, the perimeter of the pupae (projected areas of dorsal position) was detected, and the pixel numbers inside the outline were calculated. Second, the edge of each melanic spot was then detected, and the pixels for each spot were calculated. Third, the total spot area for all spots and the percentage of this area to the whole-body area were calculated. At least 100 individuals were prepared for spots analysis of each temperature treatment.

#### Adults

*Harmonia axyridis* f. *succinea* larvae and pupae were reared at 15, 20, 25 and 30°C (50% RH, 1,000 lx, 16L:8D) and prepared for adult sample collection. Adult samples of both sexes were prepared for melanic spots area proportions analysis (as described in section“Pupae,” Supplementary Figure S3C).

Adults of both sexes at ≥12 h post-eclosion were separated for imaging. The forewings (elytra) and hindwings were removed. Images of the elytra and top views of the wingless bodies were captured for analysis of the melanic spot area proportion. Melanic spot area proportion using the same equipment as for the pupae (section “Pupae”).

The proportions of spots on the elytra and melanin on the abdomens were calculated in ImageJ and based on the proportions of spot area pixels as described in section “Pupae.” At least 50 individuals were prepared for melanic spot analysis of each sex and temperature treatment.

To generate an average spots pattern, at least 15 separate images for each treatment were selected (adjust size and body orientation). After ensuring the following settings, the images were processed in Photoshop software (v. 13.0 × 64; Adobe, Mountain View, CA, United States): (1) set another 14 layers (including background image) of the basic images; (2) copied another 14 images in to each layer; (3) set: opacity: 100%,and fill opacity: 100% for all layers; (4) selected blend modes: screen, and obtained a preliminary overlaid image; (5) adjusted image: Brightness: -100, contrast: 30, and obtained a final image.

### Physiological Regulatory Mechanisms

#### Modification of Target Gene Expression by RNAi

*H. axyridis* f. *succinea* transcriptomes (mixed samples with eggs, larvae, pupae, and adults) were sequenced before the experiments. Transcriptome sequencing and preliminary analysis were performed by Wuhan Bioacme Corp. (Wuhan, Hubei, China; http://www.whbioacme.com; Hiseq 4000 (PE150/125); NGS; 12 Gb clean data obtained). Target gene sequences (*HaTH*, MK584934.1; *Halac2*, MN650656; *Hayellow*, MN650659; *Hatan*, MN650658; *HaADC*, MN650660; and *Haebony*, MN650657) were obtained from *H. axyridis* transcriptome sequencing (open reading frames; ORF) and uploaded. Transcriptional analysis of the target genes under different temperature treatments was performed to detect correlations. This method is described in detail in Supplementary Material S1.1. The results are shown in Supplementary Figure S1.

The dsRNAs of the target genes were prepared in a T7 RiboMAX System (Promega, Madison, WI, United States). Two targets were selected for the RNAi. The primers used in the synthesis of the two dsRNAs are listed in Supplementary Table S1. Twelve-hour, fourth-instar larvae and pupae were injected with 250 nL dsRNA (∼100–5,000 ng μL^–1^) for each of the target genes. A non-ladybird-related *GFP* gene was used as a control (Chen et al., 2016). The delivery of the dsRNAs is described in Supplementary Material S1.1. RNAi efficiency was measured by RT-Q-PCR (Supplementary Material S1.1). Effective dsRNAs were prepared for the subsequent experiments.

#### Phenotypic Observations

After dsRNA injection, the beetles were reared for pupation or eclosion. Treated individuals were prepared for the imaging of their melanin spots 12 h after ecdysis. Experiments conducted at 27.5°C. Digital images were acquired for phenotypic analysis (section “Pupae”). Adobe Photoshop CS6 (v. 13.0 × 64; Adobe, Mountain View, CA, United States) was used to remove the color channels.

#### Simulation of Dynamic Melanin Spot Changes in Pupae at Constant Temperature by Transcriptional Regulations of *HaADC* and *Haebony* Genes in the Aspartate-β-Alanine Pathway

To further confirm the critical role of genes in the aspartate-β-alanine pathway (*HaADC* and *Haebony*) in spots pattern regulation, we intended to reproduce melanin spot patterns induced by transcriptional modification (RNAi) at 32.5°C.

*H. axyridis* f. *succinea* larvae were reared at 32.5°C (50% RH, 1,000 lx, 16L:8D) to lower their melanin levels and prepared for injection with ds-*Haebony* and ds-*HaADC* (250 nL for each individual; ds-*Haebony*: 125, 250, 500, 1,000 ng μL^–1^; ds-*HaADC*: 500, 1,000, 2,000, 4,000 ng μL^–1^). A non-ladybird-related *GFP* gene was used as a control (250 nL, 5,000 ng μL^–1^).

#### Cuticle Morphology

Histological sectioning and staining (hematoxylin–eosin; H&E) of pupae and adults were performed for cuticle morphology analysis. The protocol is described in Supplementary Material S1.2.

#### Immunofluorescence and *in situ* Hybridization

The distribution of key molecules (mRNA or protein) were analyzed to prove the connection between phenotypes and the regulation of key pathways. Tyrosine hydroxylase, the key cuticular melanization protein, was prepared for immunofluorescence. *HaADC* was selected for *in situ* hybridization. The protocols are described in Supplementary Material S1.3.

#### Upstream Spot Pattern Regulation

We screened and analyzed the upstream regulations of spots formation between the pupal and adult stages of *H. axyridis*, as supplementary evidence of the regulation differences between the two developmental stages.

Three *Hox* genes (*Ultrabithorax, Ubx*, MN927185*; Abdominal-A, Abd-A*, MN927187*;* and *Abdominal-B, Abd-B*, MN927186) associated with thoracic and abdominal segment formation were screened and prepared for a pupal spot pattern control study. For the adult experiments, elytral vein lengths were measured and the *Rhomboid* (*Rho*) gene associated with vein formation was prepared for an elytral spot pattern control study. The protocols are described in Supplementary Material S1.4.

#### Measurement of Veins of Elytra With Different Melanin Level

First, the R color channel was removed to enhance the vein contrast to the background (Figure 5C-2); then we measured the length of the first vein (from the left, line a of Figure 5C-2) and the second vein (from the left, line b of Figure 5C-2) by pixels. Finally, the ratio of b to a (b/a) was calculated (Figure 5C-3).

### Statistical Analyses

Melanin spot analyses under the various thermal treatments were subjected to one-way analysis of variance in SPSS (v. 22; IBM Corp., Armonk, NY, United States). Significantly different treatment means were identified by Duncan’s test (*P* < 0.05). Immunofluorescence and *in situ* hybridization images were obtained and analyzed with CaseViewer v. 2.0 (3DHISTECH Ltd., Budapest, Hungary).

## Results

### Similar Dynamic Changes in Pupal and Adult Melanin Spots With Temperature

**FIGURE 1:**
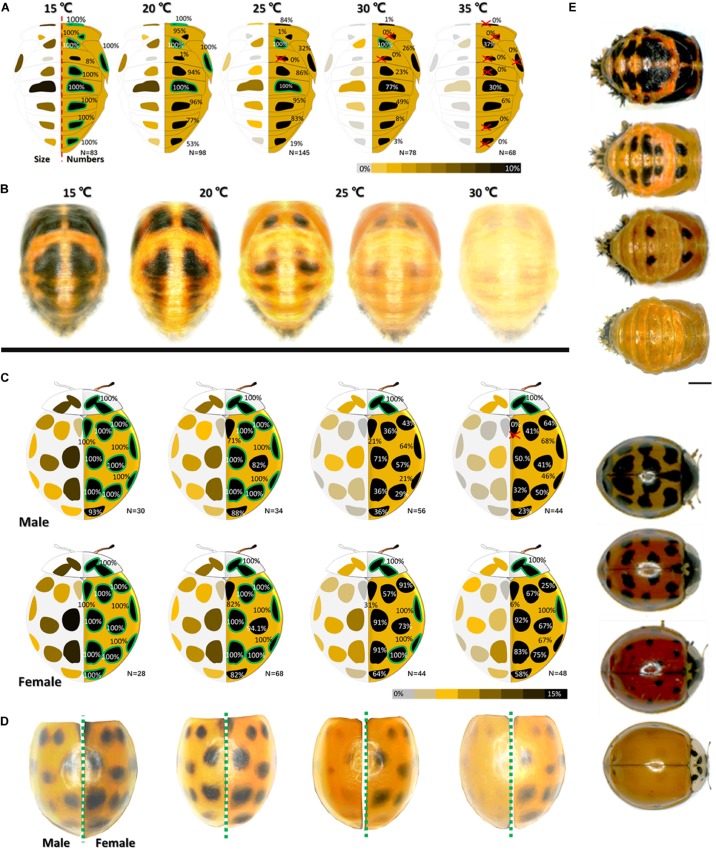
Melanin spots dynamic changes (spots size and numbers) of pupae **(A)** and **(B)** and male and female adults **(C)** and **(D)** with temperature. In **(A)** and **(C)**, the average area ratio (size) of each spot is marked by colors on the left side of each sketch. Values (one spot area/dorsal area) are indicated by a heatmap with different colors (gray (0%)-yellow-brown-black). Occurrence frequency of each spot at particular temperature observed in our samples are marked on the right of each sketch. Spots with 100% occurrence frequency at particular temperature are marked with a green edge. Those with 0% occurrence frequency are marked by a red “×.” In **(B)** and **(D)**, images (at least 15 images of similar size and positio) of pupa or elytra from the same temperature were overlaid to generated an average spots pattern. Randomly selected images of pupae and adults with different spot patterns are shown in **(E)**. See Supplementary Figures S2, S3 for more details.

In the pupae, the spot numbers and sizes decreased with increasing temperature (Figure 1). Under all temperature treatments, the largest spot was detected on the A3 segment (Figures 1A,B). In contrast, virtually no pigment spots were observed on the A1 segment; their frequency of occurrence was only 8% even at the lowest temperature (Figure 1A).

In the adults, the spot numbers and sizes also decreased with increasing temperature. The spot areas decreased with increasing temperature. Certain spots disappeared altogether (Figures 1C,D). Female adults had more and bigger melanin spots than male adults (Figure 1C). The data and statistical analyses are shown in Supplementary Figures S1–S4.

### Phenotypic Similarities and Differences Under Certain Molecular Regulatory Pathways (RNAi)

All candidate genes were successfully downregulated (Supplementary Figure S5). Four of the six genes (*Halac2, HaTH, HaADC*, and *Haebony*) presented with melanization-related functions at both developmental stages. Similar phenotypic changes were detected in both the pupae and the adults under *Halac2* and *HaTH* RNAi (dopamine regulation pathway). Different phenotypic changes were detected between the pupae and the adults under *HaADC* and *Haebony* RNAi (aspartate-β-alanine regulation pathway).

#### Similar Phenotypic Changes

**FIGURE 2:**
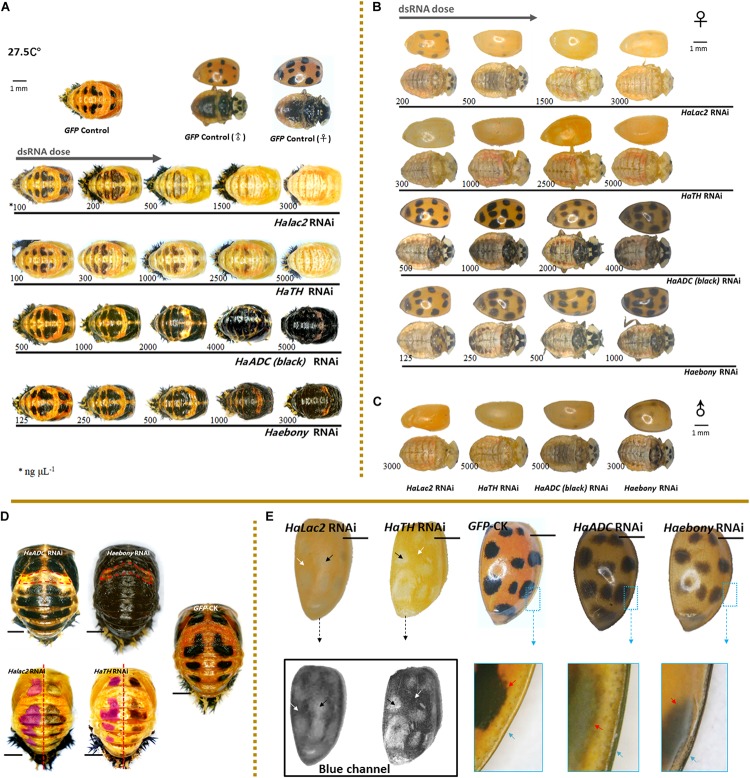
Phenotypic changes in *Harmonia axyridis* under RNAi of candidate melanization genes. Pupae **(A)** and female adults **(B)** treated with different dsRNA concentrations. Male adults treated with highest dsRNA concentration **(C)**. Experiments conducted at 27.5°C. Mortality of samples and phenotypes of corpses shows in Supplementary Figure S5. Low melanic A1 segments have been indicated using dotted boxes in *HaADC* and *Haebony* RNAi; and spots areas are marked in translucent purplish red of *HaTH* and *Halac2* RNAi **(D)**. For adult elytra, red and green channels were removed to show phenotypic changes of *Halac2* and *HaTH* RNAi **(E)**; and white arrows indicate locations without any spots, black arrows indicate spot positions, red arrows indicate spot edges (inside the enlarged blue boxes). Concretions of dsRNAs are indicated under each sample, blue arrows indicate explanate elytron margins. Scale bars = 1 mm. See Supplementary Figure S4 for more details on repression of candidate genes under RNAi.

Melanization decreased with increasing dsRNA dose under *Halac2* RNAi and *HaTH* RNAi (Figure 2). These treatments induced similar phenotypic changes in both the pupae and the adults. Spot pigment (melanin) intensity diminished in response to these treatments (Figure 2D). Intermediate individuals confirmed that neither spot size nor number was affected by RNAi exposure (Figures 2A,D). Color fading was the main effect of *Halac2* and *HaTH* downregulation in the pupae.

The phenotypic changes observed in adults under *Halac2* and *HaTH* RNAi resembled those seen in pupae (Figures 2B,C,E). The melanin spots on the elytra, pronota, and abdomen were faded (Figures 2B,C). However, the numbers and sizes of the spots, even if without melanin, were not affected by any RNAi treatment. The edges of the spots were discerned with a blue channel filter (Figure 2E).

#### Phenotypic Differences Between Pupae and Adults

##### Pupae

Melanin spot numbers and sizes increased to more-or-less the same extent under both the *HaADC* and *Haebony* RNAi treatments (Figure 2D). The other parts of the cuticle remained orange. The A1 segment was the last to undergo pigmentation in response to *HaADC* and *Haebony* downregulation (Figures 2A,D).

##### Adults

The phenotypic changes in the adults under *HaADC* and *Haebony* RNAi did not involve melanin pattern alterations (Figures 2B,C). No obvious changes in spot size or number were observed under any of these treatments. Dark melanin line observed along the edge of the elytra was the only phenotypic changes. The borders of the melanin spots at the edges of the elytra were blurred. The entire elytra background turned orange-grey at the same post-emergence stage (Figure 2E).

### Simulation of Dynamic Melanin Spot Changes in Pupae at Constant Temperature

**FIGURE 3:**
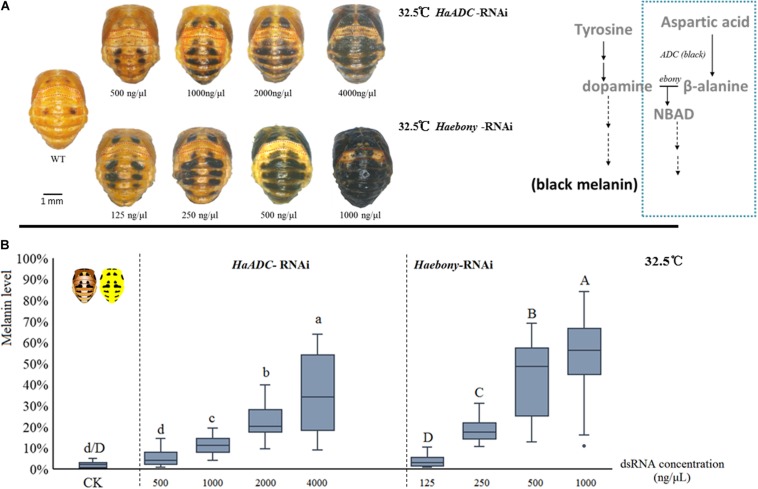
Simulation of melanin spot patterns at high temperature (32.5°C) in pupae. Melanic spot pattern **(A)** and melanin level **(B)** changes under *HaADC* and *Haebony* RNAi were analyzed. Different letters indicate statistically significant differences (analysis of variance; Duncan’s test; *P* < 0.05). A1 segments marked in red dotted box in **(A)**. Experiments were conducted at 32.5°C, at which pupal melanization is extremely low (WT of **A**). Scale bars = 1 mm.

Dynamic melanin spot changes were simulated by molecular regulation at a constant 32.5°C. Compared with the control, the insects administered with various dsRNA concentrations presented with variable melanin spot patterns. In the treated pupae, the melanin spot numbers and sizes increased with dsRNA dosage (*HaADC*-RNAi: *F* = 70.911; df = 4, 141; *P* < 0.001; *Haebony* RNAi: *F* = 109.171; df = 4, 135; *P* < 0.001; Figure 3). Enhanced pigmentation was observed for the T1 segments (pronotum) and the forewing (elytron) buds. The A1 segment was the last to undergo pigmentation under RNAi (Figure 3A).

### Distribution Analyses of the Target Molecules in Two Regulatory Pathways

**FIGURE 4:**
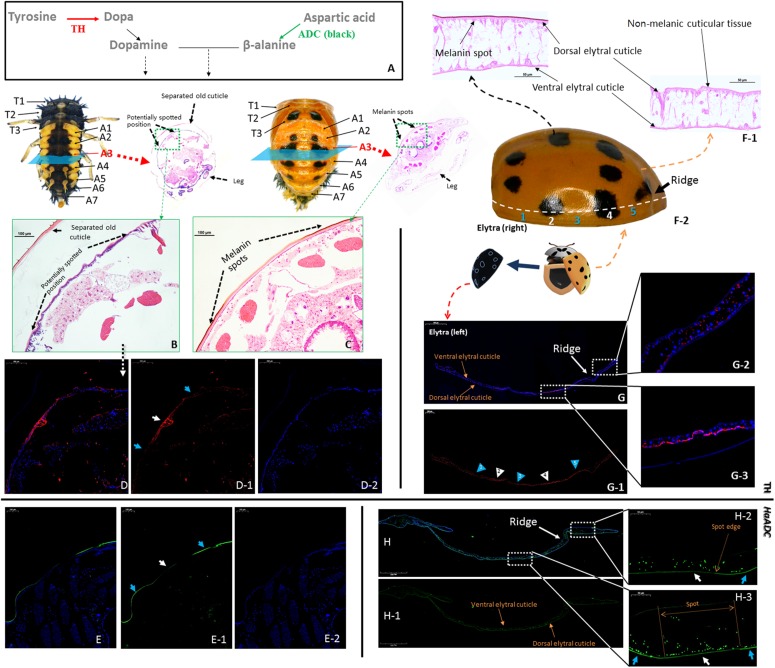
Localization of tyrosine hydroxylase (TH) and aspartate decarboxylase (*HaADC* mRNA) in the pupal epidermis (**A–E**, molecular pathway is shown in **(A)** and elytra of adults **(F–H)**. H&E-stained histological sections of the A3 abdominal segment of pre-pupae **(B)** and pupae **(C)** of *Harmonia axyridis* with spot locations indicated. Two elytra were separated from one newly emerged adult. One of the elytra was collected for H&E-stained histological sections **(F-1**) and TH immunofluorescence assay **(G)**. The other elytra was used for spot detection based on symmetry **(F-2)**. Pre-pupa **(E)** and elytra **(H)** prepared for *in situ* hybridization of *HaADC* were collected from another individuals. White arrows indicate melanin spot location (and blue arrows indicate non-melanic position. Anti-TH (rabbit) antibodies detected with Cy^TM 3^-conjugated rabbit IgG antibodies (red) **(D,G)**. Localization of *HaADC* mRNA detected by *in situ* hybridization (probes used: Supplementary Table S1). Nuclei stained with DAPI (blue). Magnifications of the melanin spots **(E-2)** and a non-melanic position **(E-1)**. White arrows with numbers in **(G-1)** indicate melanin spot locations and blue arrows indicate non-melanic positions (blue). Position numbers of **(G-2)** and **(G-3)** correspond 1:1.

Tyrosine hydroxylase and ADC (the first step in aspartate-β-alanine and tyrosine-dopamine pathways) were selected to analyze the reactions in the cuticular tissues (Figure 4A). Tyrosine hydroxylase was localized to certain locations in the new cuticle where melanin spots develop after pupation (Figures 4B–D; white arrows). Relatively weak fluorescence was detected in the unpigmented area (Figure 4D; blue arrows). Tyrosine hydroxylase distribution matched that of the melanin spots on the elytra of newly-emerged adults (Figures 4F–G). *HaADC* mRNA distribution in the cuticle matched that of TH protein. Relatively stronger fluorescence was noted for the unpigmented position (Figure 4E). There was no clear *HaADC* distribution specificity in the elytra. However, comparatively weaker fluorescence was observed in the area with melanin spots (Figure 4H). *HaADC* mRNA was detected only in the in dorsal epidermis. In contrast, no fluorescence was measured in the ventral elytral cuticle (Figure 4H).

### Upstream Regulation of Spot Formation

#### Roles of Selected Hox Genes in Spot Patterns of Pupae

**FIGURE 5:**
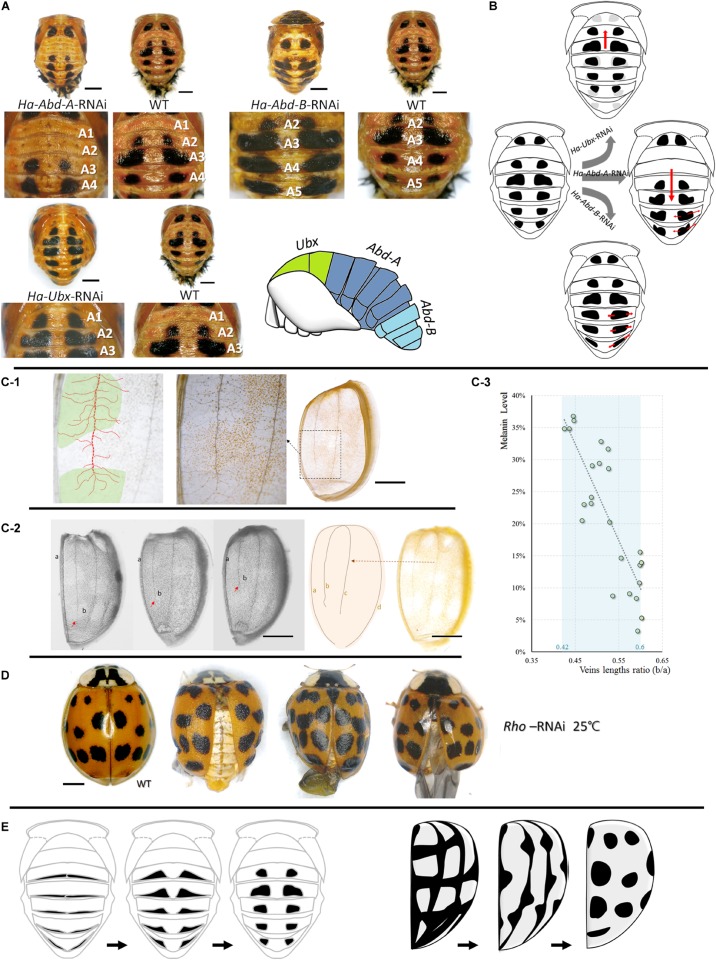
Upstream regulations of spots formations in pupae and adults. Cuticular spot pattern changes under selected *Hox* genes (*Ha-Abd-A, Ha-Abd-B* and *Ha-Ubx*) RNAi of *Harmonia axyridis* pupae **(A,B)**. Connections between elytra spots and veins **(C,D)** and the hypothesis regarding the transformation of melanic tissues on abdominal cuticle (pupa, left) and elytra (adult, right) into spots-shape during adaptation process **(E)**. More branches observed in spot positions; dot lines in red indicated mean vein and branches, and spot positions are marked in green **(C-1)**. The ratio of the second vein (b) and the sutural vein (a) is relatively higher in elytra with low melanization **(C-2,C-3)**; red arrows indicate the lengths of second veins and are different among elytra. *Rhomboid* gene (*Ha-Rho*) downregulation caused shrinkage and folding of elytra, and enlarged spots **(D)**. The different original hypothesis of melanin spots of dorsal cuticle (pupa) and elytra (adult) are shown in **(E)**. Scale bars = 1 mm.

Pupal spot patterns were strongly influenced by the selected *Hox* (*Ha-Ubx, Ha-Abd-A*, and *Ha-Abd-B*) RNAi in the thoracic and abdominal segments (Figure 5A). Spots were detected in the A1 segment (which is normally devoid of spots) and decreased in T3 underwent *Ha-Ubx* RNAi. The spots on the A2 segment were enlarged. *Ha-Abd-A* downregulation caused the spots on the A2 segment to disappear. The spots on the A3 segment shrank while those on the A4 segment enlarged. *Ha-Abd-B* downregulation enlarged the spots on segments A3, A4, and A5.

#### Association Between Elytra Veins and Spot Patterns

The four main elytra veins had more branches in the melanized (spotted) areas than the unspotted areas (Figure 5C). The ratios of the second veins (b) to the sutural veins (a) are relatively higher in elytra with low melanization. The melanin level was significantly negatively correlated with b/a vein length ratio (Pearson correlations = -0.843; *P* < 0.001; Figure 5C). Downregulation of the vein formation-related gene *Ha-Rho* resulted in elytra shrinkage and folding and spot enlargement (Figure 5D).

## Discussion

Both the pupae and adults of *H. axyridis* display similar phenotypic plasticity when they developed at diverse temperatures. To determine if these phenotypic modifications in response to ambient temperature are sharing a same regulation system or not, we examined whether pupae and adults exhibit similar responses to temperature changes and whether their thermal phenotypic plasticities are regulated by similar molecular regulatory mechanisms. Here, both pupal and adult *H. axyridis* f. *succinea* displayed similar morphological structures dependent on temperature during development. However, the reaction mechanisms occurred in different body parts (dorsal cuticle and elytra) and developmental stages (pupa and adult) and were partly regulated by different molecular pathways (the aspartate-β-alanine pathway only functions in pupae). This superficially similar phenotypic adaptation among different body parts in *H. axyridis* enables the insect to cope with environmental pressure and abiotic stress.

The multicolored Asian ladybird *H. axyridis* presents thermally-induced cuticular color pattern plasticity in its pupal and adult stages (Michie et al., 2010, 2011). The color patterns of *H. axyridis* and other ladybird species may warn predators (Daloze et al., 1994; Dolenska et al., 2009; Prùchova et al., 2014), mate choice (Osawa and Nishida, 1992; Ueno et al., 1998) and enable it to contend with ambient temperature change (Trullas et al., 2007). Thermally controlled melanism has been reported for birds, snails, and other insects (Roland, 1982; Kingsolver, 1987; Lecompte et al., 1998; Gibert et al., 2000; Solensky and Larkin, 2003; Sharma et al., 2016; Galván et al., 2018). Dark cuticular pigmentation enables beetles to absorb heat rapidly at low temperatures, whereas low pigmentation levels reduce thermal absorption at high temperatures. Infrared thermal imaging confirmed these conclusions (Supplementary Figure S2A). The modification of cuticular melanization is an effective strategy for thermal adaptation.

Both the pupae and adults could regulate cuticular melanization level by controlling the numbers and sizes of their spots naturally for thermal adaptation. Various tissues exhibiting thermosensitive melanic reaction were usually attached to the external integument and cover the body in different stages. The adult abdominal dorsal cuticle was covered by the elytra and lost its thermal sensitivity (Supplementary Figure S4E). This finding corroborates the hypothesis that melanic reaction, manifested by the regulation of spot size and number, is used for thermal adaptation in *H. axyridis*. Pupae and adults exhibited similar melanic responses to temperature changes in the form of changes to their spot patterns.

Melanization is the biochemical basis for this phenotypic plasticity (Suderman et al., 2006; Noh et al., 2016). Dopamine pathway was similar for both developmental stages. The candidate melanization genes undergo dynamic transcriptional changes at contrasting temperatures (Gibert et al., 2016, 2007). Knockdown of the dopamine pathway genes strongly influenced pigmentation (darkness) in both the pupae and the adults, but their spot shapes and numbers remained unaffected. These findings verified that candidate genes encoding melanin accumulation were functionally similar at both developmental stages and generated substrates for downstream dark pigmentation. However, they did not control either spot size or spot number.

The genes of the aspartate-β-alanine pathway regulated cuticular melanic spot distribution patterns in the pupae but not in the adults of *H. axyridis*. Knockdown of these genes resulted in melanin spot enlargement and number increase in the pupae. We propose that the aspartate-β-alanine pathway is the key regulatory pathway for pupal cuticular melanin spot deposition in *H. axyridis.* Our spot simulation experiment strongly supports this conclusion. The two relevant genes in this pathway were upregulated prior to pupation in response to temperature increases (Supplementary Figure S1). However, knockdown of the genes degrading melanin did not affect the elytral spots in adult *H. axyridis*. Thus, we conclude that the superficial spot patterns of pupal and adult *H. axyridis* could be regulated by different molecular pathways.

Tyrosine hydroxylase and ADC are the key enzymes upstream of the dopamine regulation pathway and the aspartate-β-alanine regulation pathway, respectively. The distribution of TH and ADC differed between the adults and the pupae (pronota: Supplementary Figures S7, S8). In the pupal cuticle, fluorescence difference between TH distribution between the melanic and non-melanic tissues were observed, while *HaADC* fluorescence between the melanic and non-melanic tissues of the elytra was generally at the same level. This result supported our experiment that modifying *HaADC* and *Haebony* did not enlarge the elytral spots. *HaADC* had no positional specificity in the elytral cuticle cells. This finding was also supported by the observation that the aspartate-β-alanine pathway had negligible effect on the elytral spots.

Early pigmentation indirectly indicated the regulatory differences between the developmental stages of *H. axyridis* in terms of color pattern formation. In the early pigmentation phases, the newly-emerged adults exhibited asynchronous melanization among their various organs (pronotum and elytra; Supplementary Figures S10B,C). In contrast, pupal spot formation was almost synchronized among cuticle of thorax, abdomen and wings (Supplementary Figure S10A). Transcriptional analyses indicated that *HaADC* and *HaTH* were strongly upregulated in the pronota, elytra, and abdominal cuticle during pupal and adult development (Supplementary Figure S9). These results support our expectation that pupal and adult spot patterns were regulated differently.

Regulatory differences in color pattern formation were also observed among various *H. axyridis* color forms. Compared with the orange-colored (f. *succinea*) individuals, the genetically dark background forms, f. *conspicua* and f. *spectabilis*, also have pupal cuticles with an orange background; their melanization decreases with increasing temperatures similar to that observed in the orange background form (*succinea*; Supplementary Figure S2E). Thus, the pupal melanic modification system is conserved among the various *H. axyridis* forms determined by the pannier gene (Gautier et al., 2018), while their adult counterparts have entirely different elytra. Therefore, cuticular melanization in pupal and adult elytra might be controlled by different regulatory systems.

During adaptation, pupal abdominal and adult elytral spots may be synthesized from various melanic organs/tissues precursors. The results of our experiment involving the upstream regulation of spot patterns supported this speculation. In the pupae, the thoracic and abdominal spots could be changed in the forward or reverse direction under selected *Hox* RNAi (*Ha-Ubx, Ha-Abd-A*, and *Ha-Abd-B*; Figure 5B). These *hox* genes regulate segment formation (T2, T3 and A1-A7; Supplementary Figure S11; Hughes and Kaufman, 2002). Cuticular spots change under transcriptional *Ha-Ubx* and *Ha-Abd-A* regulation in insects (Gibert et al., 2007). We believe the pupal spots are transformed from the circular melanic regions at the edges of each segment (Figure 5E). In contrast, elytral spots were strongly associated with elytral second vein length and position along the apical–basal axis of wings. *Ha-Rho* downregulation enlarged elytral spots. This effect corroborates the connection between the elytral veins and spots. We proposed that the elytral spots formation might be connected to the elytral veins, but still need more evidences (Figure 5E). These melanic features are also found in various body parts of other insect species: wings (True et al., 1999; Shevtsova et al., 2011; Tomoyasu, 2017; Nijhout and McKenna, 2018); and abdomen (Hollocher et al., 2000; Brisson et al., 2005; Ahmed-Braimah and Sweigart, 2015; Miyagi et al., 2015). In *H. axyridis* f. *succinea*, however, they are thermosensitive and form spots. On an evolutionary timescale, environmental stress (thermostimulation and solar radiation) causes these distinct cuticular melanic areas to be similar and reacting to temperature when certain parts are exposed to these stimuli at various developmental stages.

Here, we assumed this superficially similar adaptation could occur in response to universal physical or chemical solutions, key environmental pressure, and time- or position-specific cuticle directly subjected to this pressure. Under these preconditions, similar strategies may potentially be selected to deal with environmental challenges based on a universal solution but via different molecular and physiological regulatory mechanisms.

## Conclusion

In the present study, we confirmed that there are differences in regulation of melanin pattern between pupal and adult *H. axyridis*. Our data suggest that these similar adaptation strategies in melanin level regulation enable the insect to contend with temperature stress, albeit in different body parts in different developmental stages. Similar ecological adaptation strategies might be selected in the same animal species multiple times to cope with certain natural pressures. Further evidence for this biological strategy selection in other animal species is required to confirm its universality. This work may also help to clarify *H. axyridis* adaptation to its ambient environment and explain the observed variations in the distribution patterns of its melanin spots.

## Data Availability Statement

The datasets generated for this study can be found in the HaTH, MK584934.1; Halac2, MN650656; Hayellow, MN650659; Hatan, MN650658; HaADC, MN650660; Haebony, MN650657;HaUbx, MN927185; HaAbd-A, MN927187; HaAbd-B, MN927186.

## Author Contributions

YZ and T-XL designed research. X-XW and H-GT performed research. Z-JF, Z-SC, H-SC, Y-DL, L-FS, S-QZ, and W-MY provided assistance. YZ, X-XW, and YF analyzed data. YZ, H-GT, X-XW, and T-XL wrote the manuscript.

## Conflict of Interest

The authors declare that the research was conducted in the absence of any commercial or financial relationships that could be construed as a potential conflict of interest.
