# Supplementary material for: Superficially Similar Adaptation Within One Species Exhibits Similar Morphological Specialization but Different Physiological Regulations and Origins
Source: Front Cell Dev Biol. 2020 May 8;8:300. doi: 10.3389/fcell.2020.00300 (PMC7225305; doi:10.3389/fcell.2020.00300)
Supplement: Supplementary file 1 [file Data_Sheet_1.pdf]

Supplementary Information

Superficially similar adaptation within one species exhibits similar morphological specialisation but different physiological regulations and origins

[1]Yi Zhang[†]; [1]Xing-Xing Wang[†]; [1]Zhu-Jun Feng; [1]Hao-Su Cong; [1]Zhan-Sheng Chen; [1]Yu-Dan Li; [1]Wen-Meng Yang; [1]Song-Qi Zhang; [1]Ling-Feng Shen; [1]Hong-Gang Tian; [1]Yi Feng; and [1]Tong-Xian Liu

[1] Key Laboratory of Integrated Pest Management on Crops in Northwestern Loess Plateau, Ministry of Agriculture, Northwest A&F University, Yangling, Shaanxi, 712100, China

S1. Materials and Methods

S1.1. Transcriptional analysis and RNAi

Transcriptional analysis

Sequences of the target genes (*HaTH, Halac2, Hayellow, Hatan, HaADC,* and *Haebony*) were obtained from *H. axyridis* transcriptome sequencing (ORF) and uploaded. To analyze differential expression of the target genes at various rearing temperatures, f. *succinea* larvae were reared at 15, 17.5, 20, 22.5, 25, 27.5 and 30 °C (50% RH, 1,000 lx, 16L:8D). They were then prepared for transcriptional analysis. The ladybird beetle samples were frozen in liquid nitrogen immediately after collection. Integuments of the pre-pupae (fourth larvae) were collected for cuticle-specific expression analysis of the target genes. RNA was extracted from the collected samples with RNAiso Plus (Takara, Tokyo, Japan). The cDNA was synthesized with a PrimeScript™ RT reagent kit and a gDNA Eraser (Takara, Tokyo, Japan). Three individuals (at least) were used for one biological replicate and three replicates were prepared for each temperature treatment.

Quantitative real-time PCR (qRT-PCR) was performed with SYBR® Premix Ex Taq™ II (Takara, Tokyo, Japan) in an IQ-5 system (Bio-Rad Laboratories, Berkeley, CA, USA). Primers were designed with Primer-BLAST from NCBI (https://www.ncbi.nlm.nih.gov/tools/primer-blast/index.cgi?LINK_LOC=BlastHome). The primer sequences are listed in Table S1. *HaRpl3* was selected as a reference gene for qRT-PCR.

Delivery of dsRNAs

A Nanoject II Auto-Nanoliter Injector (Drummond Scientific Co., Broomall, PA, USA) was assembled with a glass needle made from a glass capillary (3.5 in 3-000-203-G/X micropipettes; Drummond Scientific, Broomall, PA, USA) using a micropipette puller (P-97 micropipette puller, Sutter Instrument, Novato, CA, USA; pulling program: pull = 100; VEL = 100; time = 100) as an injector. The target and control dsRNA solutions were separately injected into the larval ventral abdomen through the fissure at the dorsal intersegmental membrane between abdominal segments. The larvae were placed on a 35-mm plastic dish, fed with A. pisum on a *V. faba* leaf, and reared at 27.5 ± 1°C (16L:8D, 50% RH) until pupation for phenotypic analysis. At least 15 individuals were injected for RNAi each time.

Measurement of RNAi efficiency

Beginning at 1 d after dsRNA injection, the ladybird beetle samples were collected before pupation. Transcript levels were measured as described above using three independent replicates (at least five individuals were used for one biological replicate).

S1.2. Cuticle morphology analysis

HE staining

Ten individuals were prepared for Histological sectioning and staining. Haematoxylin-eosin (HE) staining was performed on the third abdominal (A3) segments of the pre-pupae and pupae. The samples were fixed overnight in 10% (w/v) buffered formalin, dehydrated, embedded in paraffin, and sectioned. Slides were prepared by soaking twice in xylene for 20 min, twice in 100% alcohol for 5 min, and 75% (v/v) alcohol for 5 min. The slides were then rinsed with water. The slides were immersed in hematoxylin solution for 3–5 min and rinsed with water. The sections were differentiated with acid alcohol, rinsed again, stained with ammonia solution and washed in slowly running tap water. The sections were then stained in eosin using 85% (v/v) alcohol for 5 min, 95% (v/v) alcohol

for 5 min, and eosin for 5 min. The sections were dehydrated, mounted thrice with 100% alcohol 5 min, twice with xylene for 5 min, and finally in colorless hyaloid resin. Digital images of the sections were acquired with a Nikon DS-Ri1 (Nikon, Tokyo, Japan) coupled to a Nikon 80i microscope system (Nikon, Tokyo, Japan) and Nis-Elements software v. 3.22.14 (Build 736, Nikon, Tokyo, Japan).

S1.3. Immunofluorescence and *in situ* hybridization

Ten individuals (each) were prepared for immunofluorescence and in situ hybridization.

Transverse sections at the A3 segment of the pre-pupae and pupae, and elytra of newly emerged adults were stained with anti-rabbit TH IgG antibody. The transverse sections were placed in a microwave oven (medium heat, 8 min---pause, 8 min --- medium-low heat, 8 min) with a repair kit filled with EDTA antigen retrieval buffer (pH 8.0). After cooling, the slides were placed in phosphate-buffered saline (PBS; pH 7.4) and washed thrice on a decolorizing shaker for 5 min each time. After drying, a circle was drawn around the tissue with an Imm-Edge pen. An Auto-Fluo Quencher was added to the circle for 5 min and it was rinsed with running water for 10 min. The tissue was then incubated with bovine serum albumin (BSA) for 30 min inside the circle. The primary antibody was mixed with PBS on a tissue section and the sections were incubated at 4ºC overnight. The slides were placed in PBS (pH 7.4) and washed thrice for 5 min each time. Secondary antibody covered with the corresponding species of primary antibody was added to the circle and incubated at room temperature for 50 min. The slides were placed in PBS (pH 7.4) and washed thrice for 5 min each time. After drying, DAPI stain was added and the slides were incubated at room temperature for 10 min. The slides were then placed in PBS (pH 7.4) and washed thrice for 5 min each time. The sections were dried and sealed with an anti-fluorescence quenching capsule. Images were photographed under a Nikon microscope (Nikon ECLIPSE CI and Nikon DS-U3, Nikon, Tokyo, Japan).

For *in situ* hybridization, transverse sections of the A3 segment of the pre-pupae were prepared as described previously. All devices and objects were operated under RNase free conditions. At the stain/hybridization stage, the samples were hybridized with *HaADC* probe (8 ng µL-l; Supplementary Table S1) in hybridization buffer (50% (v/v) formamide/5X saline sodium citrate buffer (SSC)/yeast RNA at 200 µg mL-1/heterologous DNA at 100 µg mL-1/0.1% (v/v) Tween-20) for 12 h at 37ºC. The samples were then washed, dried, stained with DAPI, and incubated at room temperature for 8 min. The sections were dried and sealed. The hybridized probes were detected (FAM, 515-555 nm, green) under a Nikon microscope (Nikon ECLIPSE CI and Nikon DS-U3, Nikon, Tokyo, Japan).

S1.4. Upstream regulations of spots patterns

In pupa

After a series of pre-experiments, three *Hox* genes (*Ultrabithorax, Ubx*; *Abdominal-A*, *Abd-A*; and *Abdominal-B*, *Abd-B*) those related to the thoracic and abdominal segments formations were finally screened and prepared for pupal spots pattern control study. Segments of sequences of *Ha-Ubx, Ha-Abd-A* and *Ha-Abd-B* were obtained from *H. axyridis* transcriptome sequencing and used for dsRNA *in vitro* synthesis (S1.1). The RNAi were performed at pre-pupa stage under 27.5 ± 1°C (16L:8D, 50% RH) (S1.1). After dsRNA injection, the beetles were reared until pupation. Pupae were prepared for image acquisition of their melanin spots. At least 15 individuals were injected for RNAi each time.

In adult

Measuring the length of elytra veins. The transparent elytra were collected immediately after molting from new emerged adults. Images were captured by SDPTOP-SZN71 microscope system (Sunny, Hangzhou, Zhejiang, China). The lengths of first vein and the second vein form elytral suture were measured by ImageJ (v. 1.51j8, Wayne Rasband, NIH, Bethesda, MD, USA). The objects were zoomed in and magnified for more details of elytra vein and captured. Thirty individuals were prepared for vein analysis and 25 valid data were finally collected.

After a series of pre-experiments, *Rhomboid* (*Rho*) gene that related to the veins formation was finally prepared for elytra spots pattern control study. Sequences segment of *Ha-Rho* was obtained from *H. axyridis* transcriptome sequencing and used for dsRNA *in vitro* synthesis (S1.1). The double injection RNAi were performed at pre-pupa and pupa stage under 27.5 ± 1°C (16L:8D, 50% RH) (S1.1). After dsRNA injections, the beetles were reared till ecdysis. Adults were prepared for image acquisition of their elytra melanin spots. At least 15 individuals were injected for RNAi.

S2. Tables and Figures

Table S1. List of primers utilized in this study

	Forward (5' to 3')	Reverse (5' to 3')	PCR type
ds-*HaTH*-1	T7*+GGTTTGCTAAAAACTCGCAA	T7+GCATCATCGACTAAAGAACG	dsRNA
ds-*HaTH*-2	T7+AACACAAGCGATAAAAGACG	T7+GGACTTTTGCGGGTAATTTT	dsRNA
ds-*Halac2*-1	T7+GGCATTTTGCAAGAACATCC	T7+GGGTTGTTGAACTTCGTCTA	dsRNA
ds-*Halac2*-2	T7+GGAAAGGGTCAATTCAGAGA	T7+AAACGGAAGCGAATGAATTG	dsRNA
ds-*Hatan*-1	T7+ACGATAAGTTGTGGAGAACA	T7+TGATCCAAACTCAAGGCTAT	dsRNA
ds-*Hatan*-2	T7+CTACGATGCAACTCTCAACA	T7+CCGCATAGCATAAAGAGGTA	dsRNA
ds-*Hayellow*-1	T7+CGAATTGGCCTATGGTATGA	T7+AGTTGGACACCATAATCGTC	dsRNA
ds-*Hayellow*-2	T7+GGTATTGGAAACACCACTCA	T7+CAAGGACATACCGAAAACAC	dsRNA
ds-*Haebony*-1	T7+CTGGAGATTTTGCACGATTG	T7+TCTTTCCATTGACCAGAAGC	dsRNA
ds-*Haebony*-2	T7+TATACCGCTTCTGGTCAATG	T7+AGCTGCTATGAAATCGCTAA	dsRNA
ds-*HaADC*-1	T7+CGCTTAGTTTTGTTCACCTC	T7+GAGATGTCTATGCTTCTGGG	dsRNA
ds-*HaADC*-2	T7+AGGAGATGAGGAGGATTGTT	T7+CAAAGGTCAGCAATTTCGTT	dsRNA
ds-*Ubx*	T7+GGCAATCATGAACTCCTACT	T7+ATTTTGTTGGCTTTCTGACG	dsRNA
ds-*Abd-A*	T7+GCGCGTTTAGTGTCTTCGAC	T7+TAGGGGTACATCCTGGAGGC	dsRNA
ds-*Abd-B*	T7+CCCGACGTACTACAACCTGG	T7+GGTGTAGGGAGAATACCCGC	dsRNA
ds-*Rho*	T7+TACTACTCGGTGGTCAGGGG	T7+AAAGAGCGTAGACACCACCG	dsRNA
ds-*GFP*	T7+CGACGTAAACGGCCACAA	T7+GTCGTCCTTGAAGAAGATGGT	dsRNA
Q-*HaTH*	TTGGTTGGTGACGATAACAT	TGGATCACCGTATTTGAAGG	Q-RT-PCR (E=86.6%)
Q-*Halac2*	TCTTGAGATACGCTAGAGGT	TCATCTCTTTGCTCGTTACA	Q-RT-PCR (E=117.9%)
Q-*Hatan*	AAGGAAGATGGAACGTCAAA	GGCACTGATTGTGTTTATCG	Q-RT-PCR (E=100.5%)
Q-*Haebony*	GAAGTGGACATCAGTTCAGG	GACCAATATGTTTTCCCTCG	Q-RT-PCR (E=101.5%)
Q-*Hayellow*	GAAATCCAACGAGCTAGGAA	TTGAAGACGTTGATGCTGTA	Q-RT-PCR (E=97.8%)
Q-*HaADC*	CAAGAGGCAGAATAAGGTCA	TACGCAATCGTATCCCTAAC	Q-RT-PCR (E=102.9%)
ref-*Harps3*	GGCTACCAGAACCGAACAGAG	GTGCTATGGCGCATAATCCT	Q-RT-PCR (E=87.3%)
probe -*HaADC*	TGCAAGGAGTCCTTTGTGTTTAAGTAGG		*in situ* hybridization

*T7 sequence: 5'-taatacgactcactataggg -3'

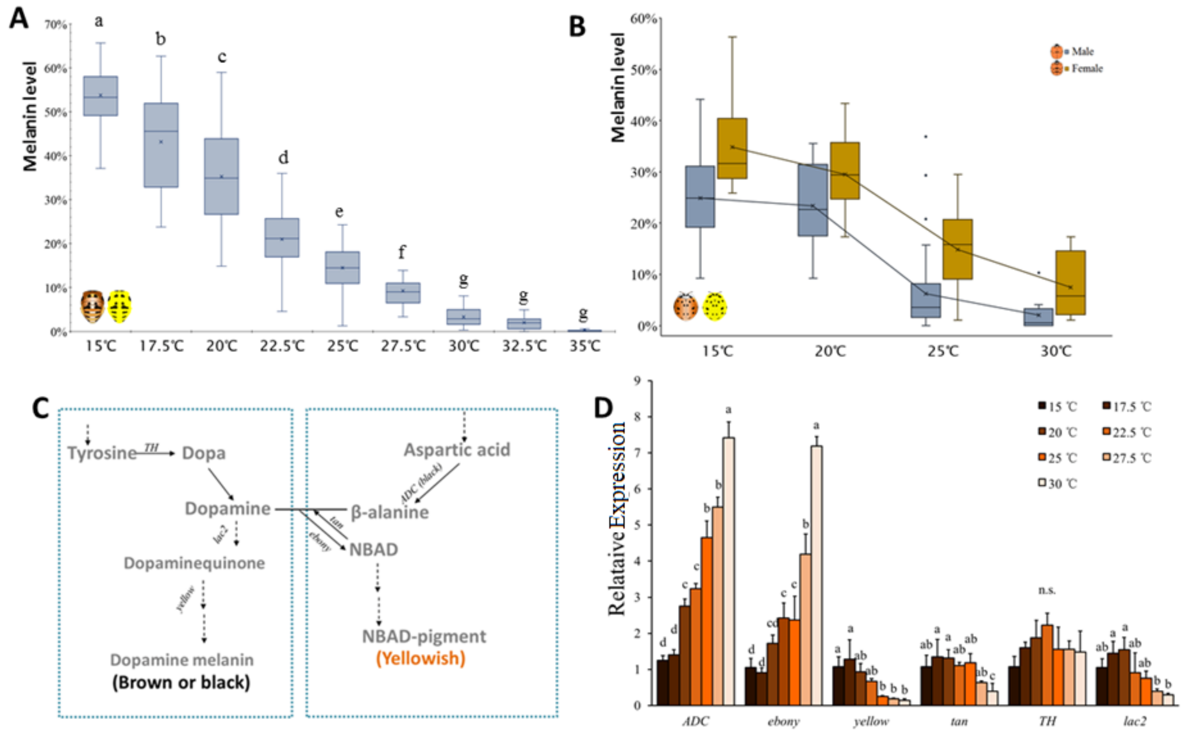

Fig. S1 Thermal variation in melanin patterns and candidate genes of melanization of *Harmonia axyridis*. Melanin area changes under exposure to various temperatures of pupae and male and female adults are shown in A and B. Molecular regulation pathway is shown in C (Noh et al. 2016). Expression levels of candidate genes at various temperatures are shown in D. Different letters in A and D indicate statistically significant differences (ANOVA; Duncan's test; $P < 0.05$); n.s. in D indicates no significant difference.

Dynamic changes in melanin spots were observed in both pupal and adult *H. axyridis*. Melanin levels in the pupal cuticle decreased with increasing temperature. The numbers and sizes of spots increased during this process ($F = 265.285$; df = 8, 348; $P < 0.001$; A). The A1 segment was pigmented only at the lowest temperatures. The melanin levels in the adults also decreased with increasing temperature. The females normally showed stronger melanization than the males under all thermal treatments ($F = 50.975$; $P < 0.001$; no interaction between sex and temperature; $P = 0.429$; B).

Of all candidate genes (C), *HaADC* (*black*) and *Haebony* were upregulated with rising temperature (*HaADC*: $F = 61.106$; df = 6, 14; $P < 0.001$; *Haebony:* $F = 30.264$; df = 6, 14; $P < 0.001$; D). In contrast, *Hayellow* was downregulated with increasing temperature ($F = 3.573$; df = 6, 14; $P = 0.023$; D). There was no strong evidence of any transcription pattern for *Hatan*, *HaTH*, or *Halac2*. No significant change in *TH* expression was observed in any treatment (*Hatan*: $F = 1.773$; df = 6, 14; $P = 0.177$; *HaTH:* $F = 0.733$; df = 6, 14; $P = 0.632$; *Halac2*: $F = 2.565$; df = 6, 14; $P = 0.069$; D).

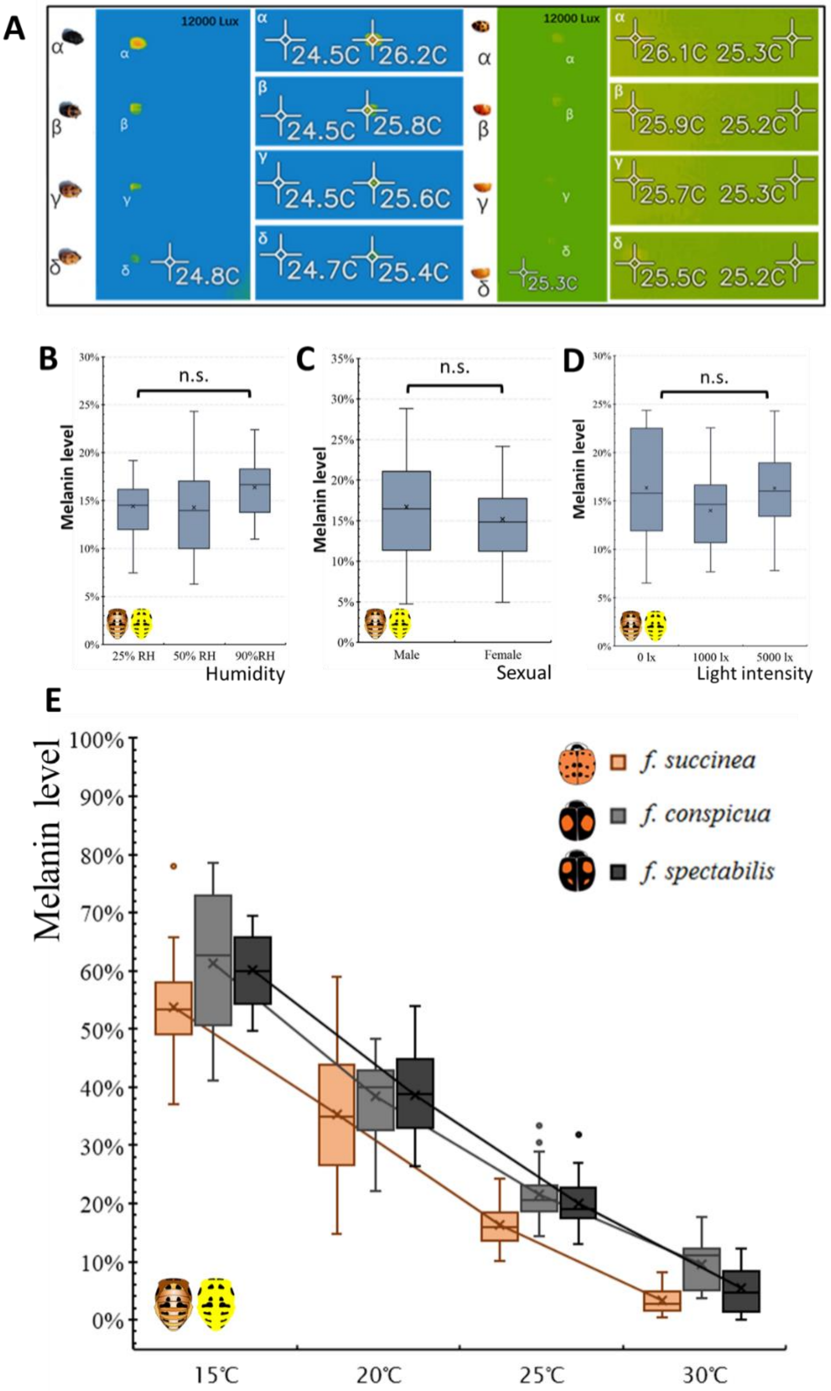

Fig. S2 The heating absorption differences of melanic pupae and adults of *Harmonia axyridis* (infrared thermal imaging, A). Pupal melanin level changes under humidity (B) regulations, sexual (C), different light intensities (D) and among different forms (elytral pattern, E) were analyzed; n.s. indicates no significant difference in values (ANOVA, Duncan's test, $P < 0.05$). In addition to temperature, other physical factors, humidity, sexual and light intensity were tested, and no significantly effects were found on melanin spots of *H. axyridis* pupa (humidity: $F = 1.532$, df = 8, 68; $p = 0.223$; sexual: $t = 1.006$, df = 72; $p = 0.318$; light intensity: $F = 2.012$, df = 2, 81; $p = 0.14$;B, C, D). Pupal melanin level of different *H. axyridi* colour forms were different. Individuals expected to have dark background of elytra (f. *conspicua;*

f. *spectabilis*) have relatively stronger melanin level in their pupal stage than individuals expected to have orange background (f. *succinea*) in all our thermal treatments ($F = 15.814$, $p < 0.001$; with no interaction between forms and temperatures, $p = 0.109$, E).

Melanin spots dynamic patterns (spots size and numbers) of pupae and adults

Humidity:

Larvae (f. *succinea*) at the third instar form rearing condition (27.5 ± 1°C, 50% RH, 1000 lux, 16L: 8D) were transferred into the low humidity condition (27.5 ± 1°C, 25% RH, 1000 lux, 16L: 8D) and the high humidity condition (27.5 ± 1°C, 90% RH, 1000 lux, 16L: 8D). Samples at the pupa stage were then collected for melanin level analysis.

Light intensity:

Larvae (f. *succinea*) at the third instar form rearing condition (27.5 ± 1°C, 50% RH, 1000 lux, 16L: 8D) were transferred into the higher light intensity level (27.5 ± 1°C, 50% RH, 5000 lux, 16L: 8D) and dark treatment (27.5 ± 1°C, 50% RH, 0 lux, 0L: 24D). Samples at the pupa stage were then collected for melanin level analysis.

Sexual

Larvae (f. *succinea*) were rearing (27.5 ± 1°C, 50% RH, 1000 lux, 16L: 8D) and prepared for sample collection. Samples at the pupa stage were then collected for melanin level analysis and rearing continued separately, the genders were identified after emergence.

Colour forms

Larvae of three colour forms (f. *succinea*; f. *conspicua*; f. *spectabilis*) were reared at four different temperatures (15, 20, 25, and 30°C; 50% RH, 1000 lux, 16L: 8D). Samples at the pupal stage were then collected for melanin level analysis.

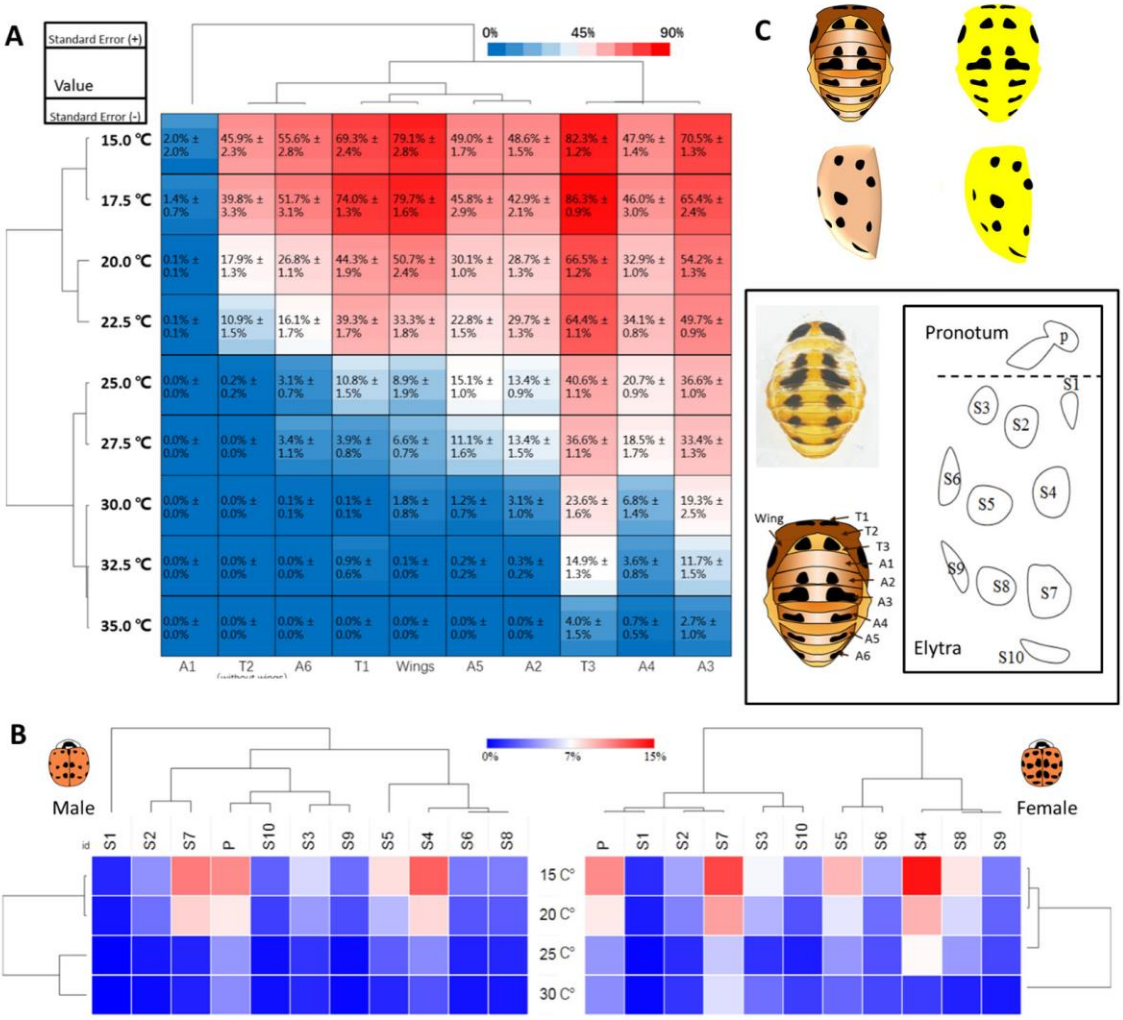

Fig. S3. Thermal melanic spots patterns of *Harmonia axyridis* pupae (A) and adult elytra (B). The spots size level of each pupal segment and elytra at various temperatures are shown in heatmap and clustered by Morpheus, different colours represent melanization level. Morpheus was used to generate heat maps for agglomerative hierarchical clustering analysis (HCA). HCA for the activity heat map was performed using the Euclidean metric with complete linkage. The calculation and naming rules of each spot are shown in C. Each segments of pupal stage exhibited similar decreasing trend during temperature rising, except for A1, which was normally un-melanic under all our thermal conditions (Fig. 3A). There are gaps detected among different temperatures between 17.5°C and 20°C, 22.5°C and 25°C (the biggest one); 27.5°C and 30°C.

Table S2. Spots occurrence frequency of each segment in pupa

Segment	Pupae<br>Temperature (samples size)<br>15°C (83)	20°C (98)	25°C (145)	30°C (78)	35°C (68)
T1	83	98	122	1	0
T2	83	92.75	2	0	0
T3	83	98	145	78	25
A1	7	1	0	0	0
A2	83	92	125	18	0
A3	83	98	145	60	20
A4	83	94	138	38	4
A5	83	75	121	6	0
A6	83	52	28	2	0
wing	83	98	46	20	0

Table S3. Spots occurrence frequency of each spot's mark* in elytra

		Male<br>Temperature (samples size)				Female<br>Temperature (samples size)			
		15°C (30)	20°C (34)	25°C (56)	30°C (44)	15°C (28)	20°C (68)	25°C (44)	30°C (48)
	Pronota	30	34	56	44	28	68	44	48
Elytra	S1	30	24	12	0	28	56	14	3
	S2	30	34	20	18	28	68	25	32
	S3	30	34	24	28	28	68	40	12
	S4	30	34	40	22	28	68	40	44
	S5	30	28	32	18	28	64	32	32
	S6	30	34	36	30	28	68	44	48
	S7	30	34	20	14	28	68	40	40
	S8	30	34	16	22	28	68	44	36
	S9	30	34	12	20	28	68	44	32
	S10	28	30	20	10	28	56	28	28

* spots marks show in Fig. S3C

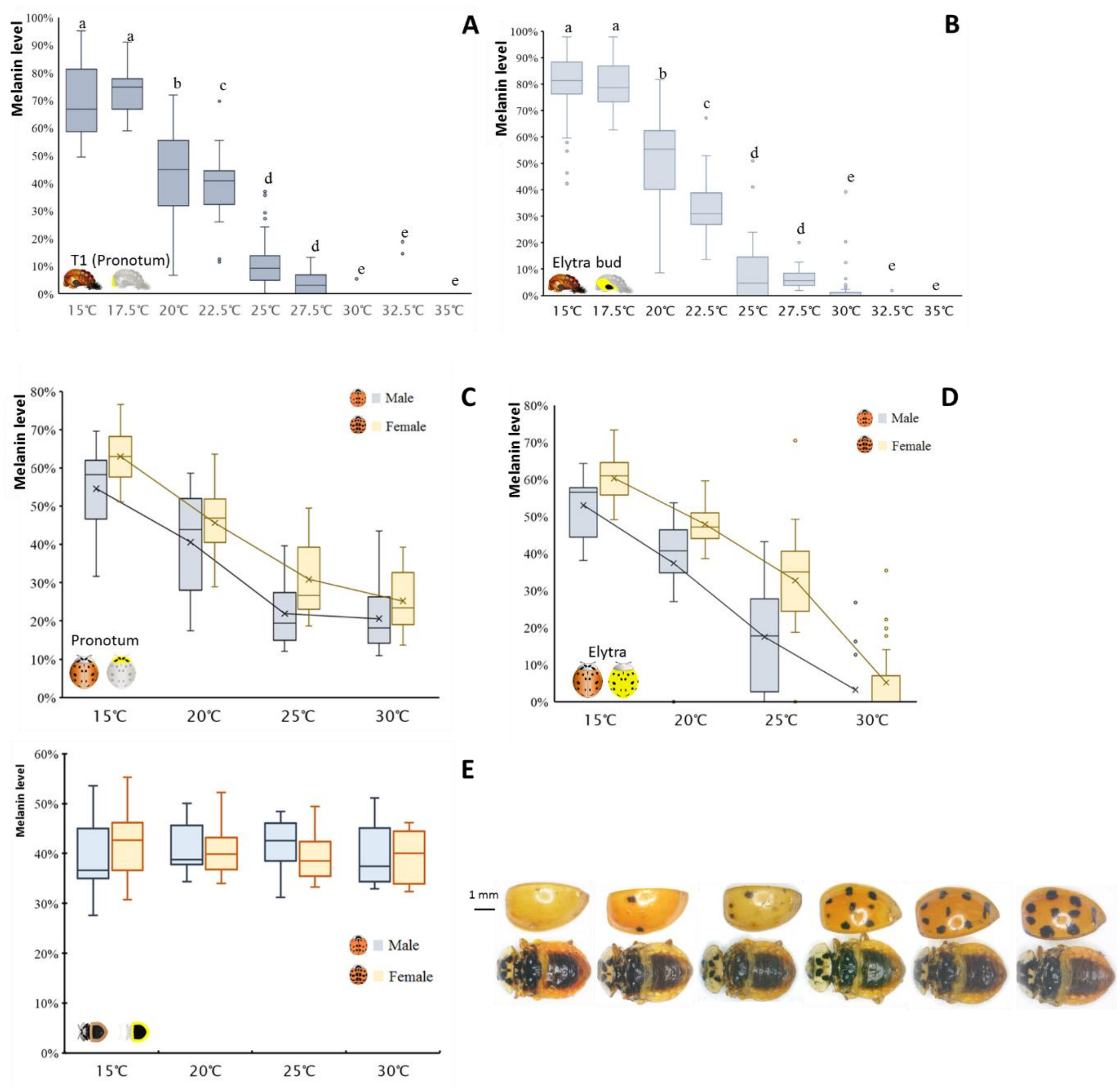

Fig. S4. Thermal melanic changes of *Harmonia axyridis* pupal and adult's pronotum, elytra and abdominal dorsal cuticle. Melanin level of pupal pronotum and elytra buds were picked and shown in A and B. Melanin level of adult pronotum, elytra and abdominal dorsal cuticle were shown in C, D and E. Different letters in A and B indicate significant differences among means (ANOVA, Duncan's test, $P < 0.05$). Scale bars of E: 1 mm. Pronotum segment and elytra exhibited similar trend under thermal conditions in pupa and adult (pronotum of pupa: $F = 406.788$, df = 8, 340; $p < 0.001$; elytra bud of pupa: $F = 371.622$, df = 8, 338; $p < 0.001$; pronotum of adults: $F = 258.743$, $p < 0.001$, no interaction between sex and temperatures, $p = 0.722$; elytra of adults: $F = 415.679$, $p < 0.001$, no interaction between sex and temperature, $p = 0.605$; A-D); but melanization level of abdominal dorsal cuticle of adults showed no change among different temperature conditions ($F = 0.722$, $p = 0.398$, no interaction between sex and temperature; Fig. E), and melanin levels between males and females were also similar ($F = 3.094$, $p = 0.082$; Fig. E).

RNAi of candidate genes for cuticular melanization.

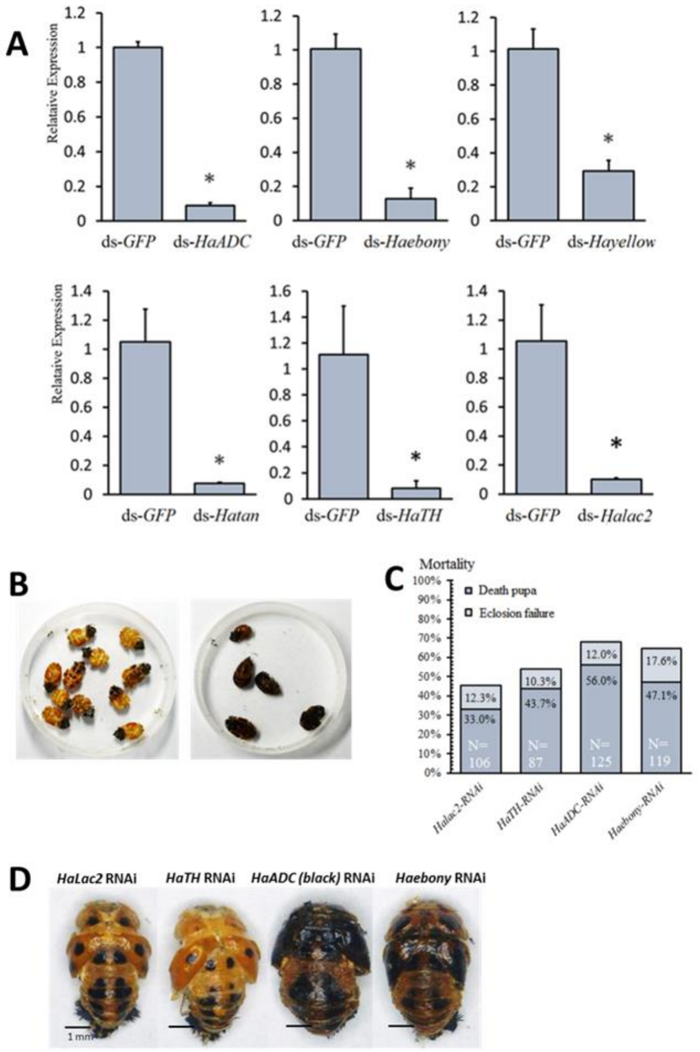

Fig. S5. RNAi of candidate genes of melanization (A), mortality of *Harmonia axyridis* during pupal stage and ecdysis under RNAi (C) and phenotypes observed of corpse (B & D). * of A indicates significant difference at $P < 0.05$ (Mann-Whitney U test). Scale bars of D: 1 mm. Comparing with ds-*GFP* control, all candidate genes were observed strong down-regulated at 24 hours after ds-RNAs injection (*HaADC*: Z = -1.993, $p = 0.046$; *Haebony: Z* = -1.964, $p = 0.05$; *Hayellow*: $t = -1.993$, $p = 0.046$; *Hatan*: $Z = -1.964$, $p = 0.05$; *HaTH: Z* = -1.964, $p = 0.05$; *Halac2*: $Z = -1.964$, $p = 0.05$; A).

Down-regulation of *Hayellow* and *Hatan* did not cause any obvious phenotypic changes in our experiments. Although previous studies reported *yellow* and *tan* were responsible for the colour pattern formation in *Drosophila melanogaster* and *Bombyx mori* (Futahashi et al, 2008; 2010; Ito et al, 2010; Gibert et al, 2016), studies also showed *yellow* RNAi had no

effect in *Tribolium castaneum* (Arakane et al; 2010). Combining with our data, it is suggested that *yellow* and *tan* might be not critical for *H. axyridis* cuticle pigmentation, and the melanic regulation system was different from that in *Drosophila* (Gompel et al 2005; Wittkopp and Beldade, 2002; 2009).

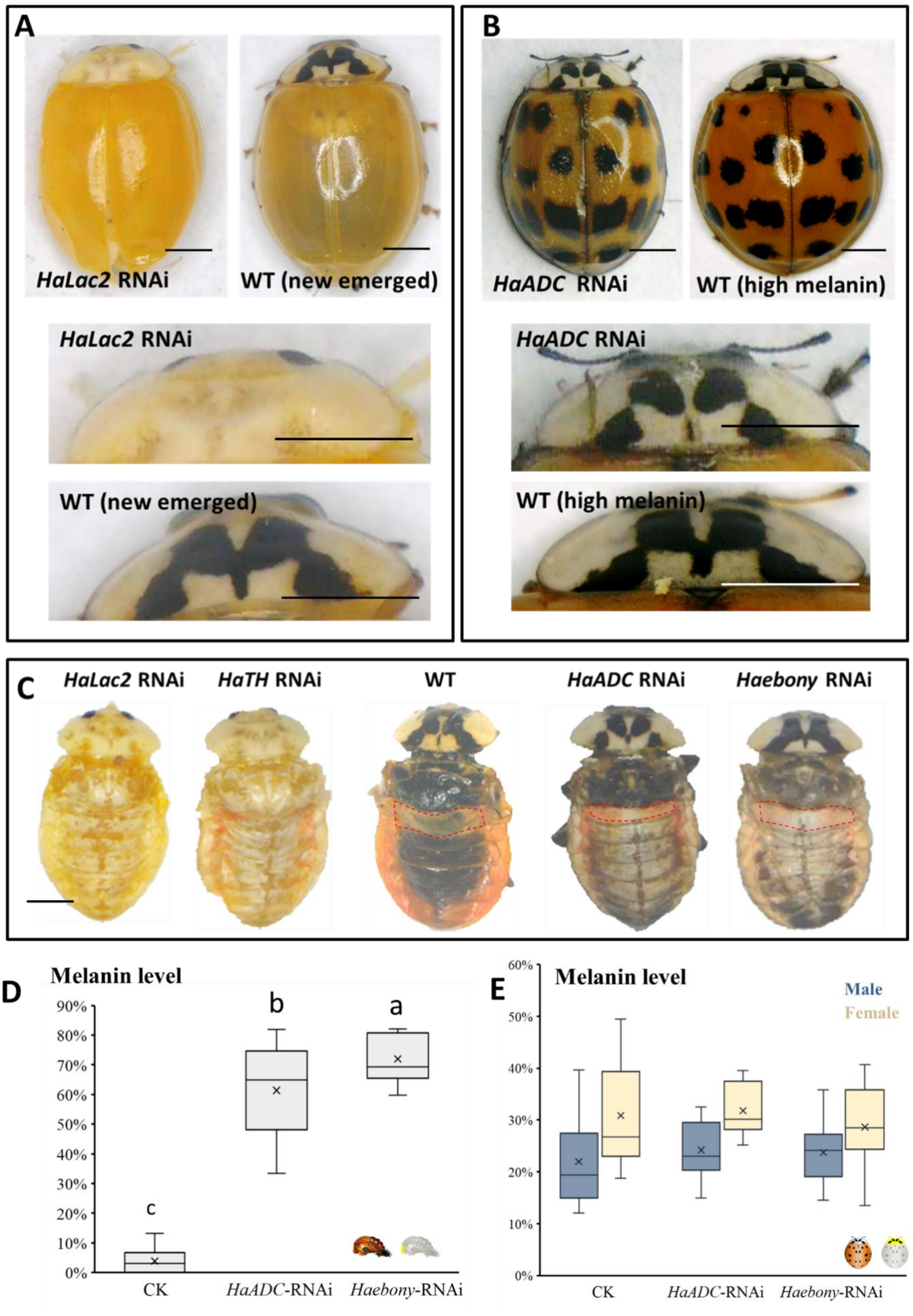

Fig. S6. Phenotypic details of pronota (A & B) and abdominal dorsal cuticle (C) in *Harmonia axyridis* adults under RNAi of candidate melanization genes. A1 segments are marked in dotted box of *HaADC* and

Haebony RNAi in C. Scale bars of A-C = 1 mm. See Figs. S3 & S8 for more details on melanization of the abdominal dorsal cuticle. Melanin level of pupae (D) and adults (male and female, E) changes under *HaADC* and *Haebony* RNAi, and different letters indicate significant differences in values (ANOVA, Duncan's test, $P < 0.05$). Melanin of pronotum could be affected and turned dark under down-regulations of *HaADC* and *Haebony* in pupa ($F = 318.743$, df = 2, 43; $p < 0.001$; D); but no change detected in melanization on it under *HaADC* and *Haebony* RNAi in adults ($F = 5.983$, $p = 0.912$, no interaction between sex and treatments, $p = 0.331$; E). Experiments conducted at 27.5 ºC, wildtype were prepared as CK.

Distributions analysis of TH and *HaADC* (mRNA) of adult pronotum

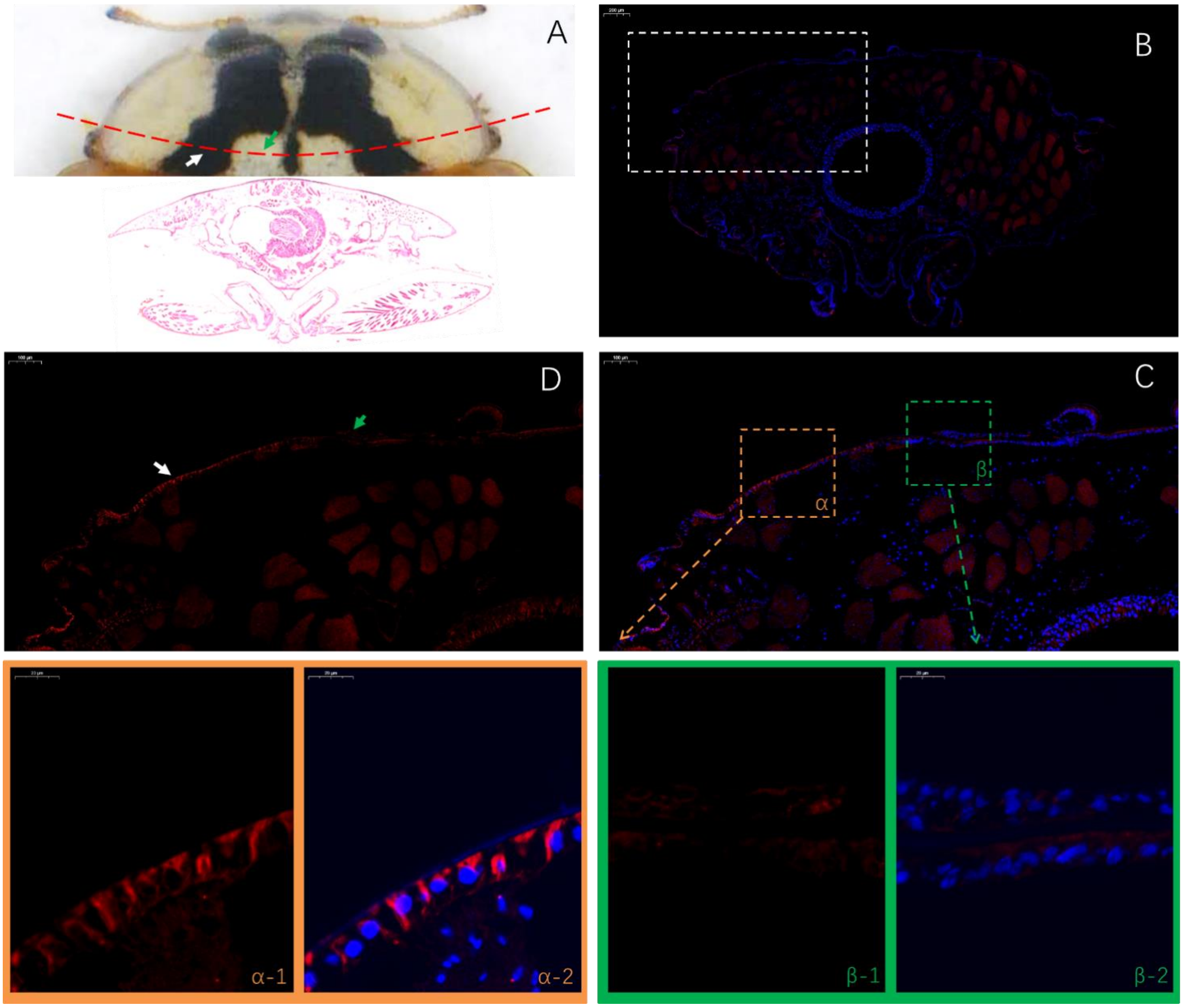

Fig. S7. Localization of Tyrosine hydroxylase (TH) in pronotum of developing adult inside of the pupae (3 days old pupa at 27.5°C). The crosscutting position was shown in A (not the same individual). Arrows of A and D indicate melanin spots location (white) and non-melanic position (green). This result indicates that TH shows a positional distribution associated with melanic spots on adult's pronotum.

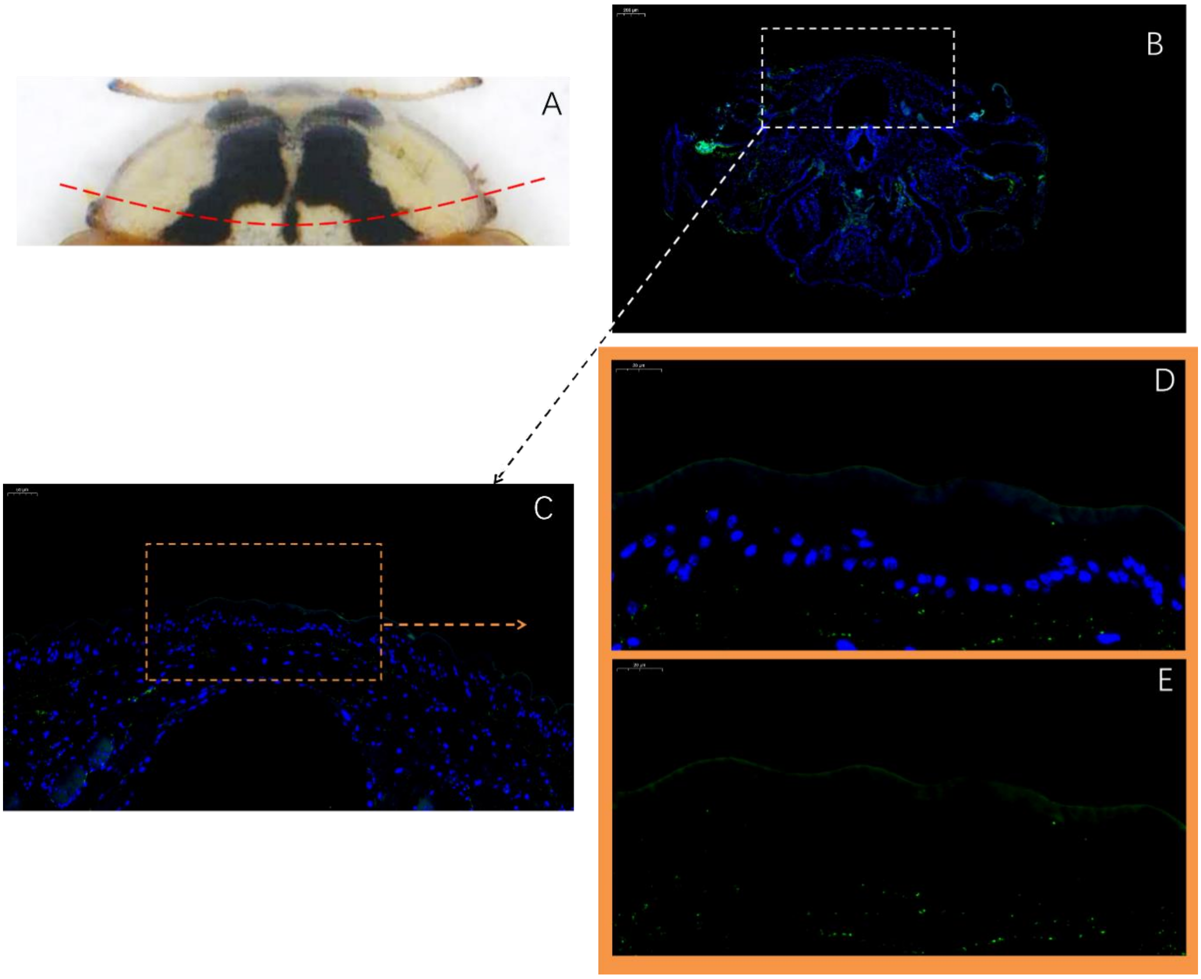

Fig. S8. Localization of *HaADC* (mRNA) in pronotum of developing adult inside of the pupae (3 days old pupa at 27.5°C). The crosscutting position was shown in A (not the same individual). Samples with different magnifications were shown in B, C, D (mixed with DAPI) and E (*HaADC* only). This result indicates that expression of *HaADC* was weak at adult's pronotum.

Transcriptional analysis of *HaADC* and *Haebony* in pronotum, elytra and abdominal dorsal cuticle.

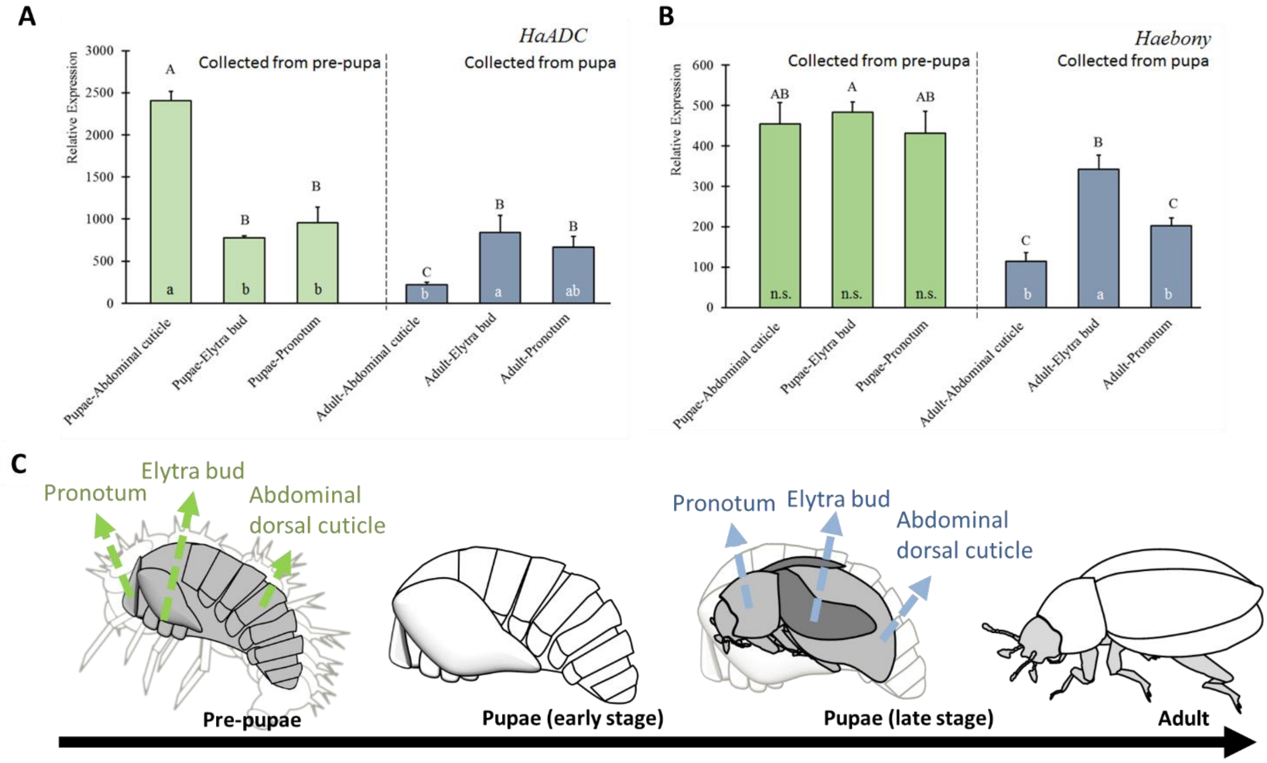

Fig. S9. Transcriptional differences of *HaADC* (A) and *Haebony* (B) in abdominal dorsal cuticle, elytra bud and pronotum for pupa and adult of *Harmonia axyridis*, respectively. The sampling method is exhibited in C. Different letters of A and B over the bars indicate significant differences in values of all bars, and lowercase letters inside indicate significant differences in values of the bars in the same colour (ANOVA, Duncan's test, $P < 0.05$); n.s. indicates no significant difference. Sample of mature adults were prepared as a control (relative expression level = 1) and the bars were not shown in graph.

Pre-pupal stage generally last for about 20 hours at 27.5°C; individuals at pre-pupa stage were reared for 18 hours (27.5 ± 1°C), and the un-pupated individuals were selected for samples collection; the incomplete pupa inside was taken out and its pronotum, elytra and abdominal dorsal tissues were dissected and collected for further transcriptional analysis.

Pupa stage generally lasts for about 3-4 days at 27.5°C; individuals at pupa stage were reared for 3 days (27.5 ± 1°C), the individuals those were still in pupa stage were selected, the incomplete adults inside were took out and pronotum, elytra and abdominal dorsal tissues were collected for further transcriptional analysis.

Results:

Comparing with mature adult bodies sample, both *HaADC* and *Haebony* in pronotum, elytra and abdominal dorsal cuticle were strongly up-regulated. Transcription level of *HaADC* was up-regulated 220~2400 fold than that of control in our analysis; and *Haebony* was up-regulated 100~480 fold (Fig. S8).

In *HaADC* expression analysis, expression of target gene up-regulated the most in the sample of abdominal dorsal cuticle, collected from pre-pupa (F = 31.92, df = 5, 12; p < 0.001, Fig. S8A). The expression patterns among different tissues (pronotum, elytra bud and abdominal dorsal cuticle) were different in samples collected from different developmental stages (samples from pre-pupa for pupa: F = 50.855, df =2, 6; p < 0.001; samples from pupa for adults: F = 5.403, df =2, 6; p = 0.046; Fig. S8A).

In *Haebony* expression analysis, expression of target gene was up-regulated stronger in the samples collected from pre-pupa (F = 15.831, df = 5, 12; p < 0.001, Fig. S8B). The expression patterns among different tissues (pronotum, elytra bud and abdominal dorsal cuticle) of pupa (for adults) were similar to those of *HaADC* expressions (elytra bud > pronotum > abdominal dorsal cuticle; F = 19.824, df =2, 6; p = 0.002; Fig. S8B). No difference observed in samples collected from pre-pupa (for pupa; F = 0.316, df =2, 6; p = 0.741; Fig. S8B).

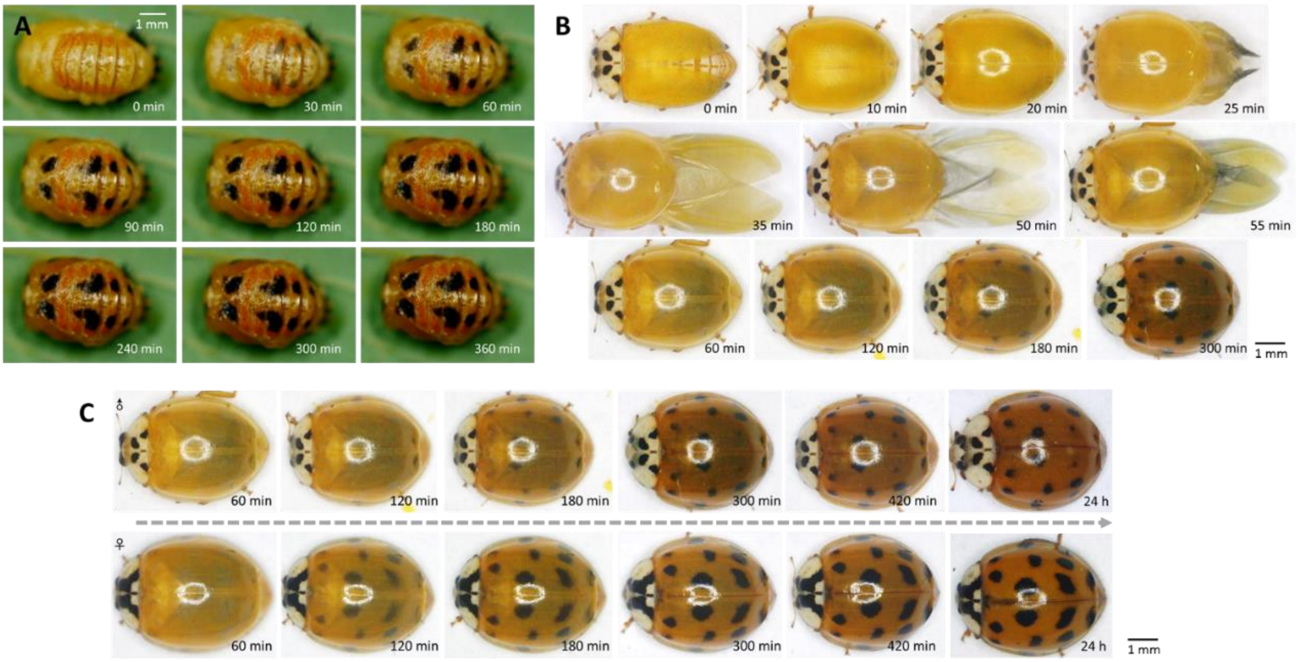

Fig. S10. Early melanic process of pupae (A) and adult (B) after pupation and emergence of *Harmonia axyridis*, respectively. Male and female adult emergence process were also compared (C). Scale bars: 1 mm.

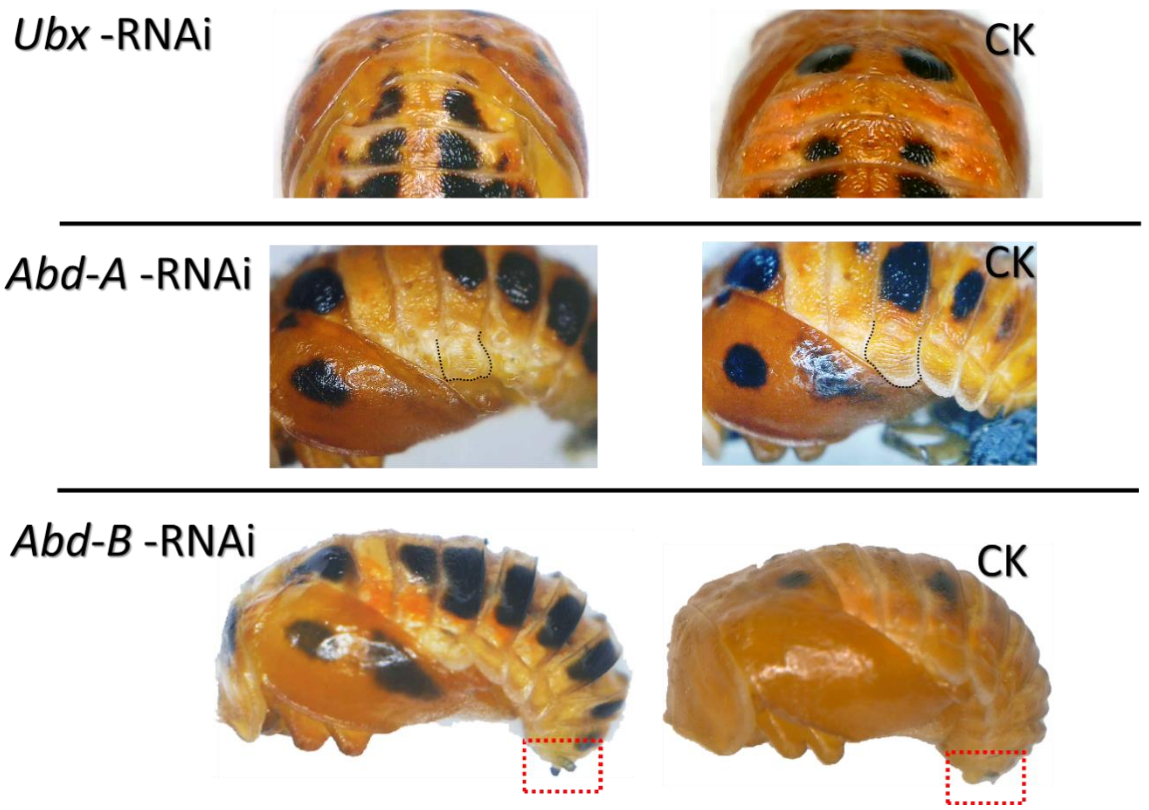

Fig. S11. Phenotypic changes in morphology of *Harmonia axyridis* pupae under RNAi of *Ubx*, *Ha-Abd-A*, *Ha-Abd-B*. Wild-type individuals (no-dsRNA treated) were selected as a CK.

References:

Arakane, Y., Dittmer, N.T., Tomoyasu, Y., Kramer, K.J., Muthukrishnan, S., Beeman, R.W. and Kanost, M.R., 2010. Identification, mRNA expression and functional analysis of several *yellow* family genes in *Tribolium castaneum*. Insect biochemistry and molecular biology, 40(3), pp.259-266.

Futahashi, R., Banno, Y. and Fujiwara, H., 2010. Caterpillar colour patterns are determined by a two‐phase melanin gene prepatterning process: new evidence from *tan* and *laccase2*. Evolution & Development, 12(2), pp.157-167.

Futahashi, R., Sato, J., Meng, Y., Okamoto, S., Daimon, T., Yamamoto, K., Suetsugu, Y., Narukawa, J., Takahashi, H., Banno, Y. and Katsuma, S., 2008. *yellow* and *ebony* are the responsible genes for the larval colour mutants of the silkworm *Bombyx mori*. Genetics, 180(4), pp.1995-2005.

Gibert, J.M., Mouchel-Vielh, E., De Castro, S. and Peronnet, F., 2016. Phenotypic plasticity through transcriptional regulation of the evolutionary hotspot gene tan in *Drosophila melanogaster*. PLoS Genetics, 12(8), p.e1006218.

Gompel, N., Prud'homme, B., Wittkopp, P.J., Kassner, V.A. and Carroll, S.B., 2005. Chance caught on the wing: cis-regulatory evolution and the origin of pigment patterns in *Drosophila*. Nature, 433(7025), p.481.

Ito, K., Katsuma, S., Yamamoto, K., Kadono-Okuda, K., Mita, K. and Shimada, T., 2010. Yellow-e determines the colour pattern of larval head and tail spots of the silkworm *Bombyx mori*. Journal of Biological Chemistry, 285(8), pp.5624-5629.

Wittkopp, P.J. and Beldade, P., 2009, February. Development and evolution of insect pigmentation: genetic mechanisms and the potential consequences of pleiotropy. In Seminars in Cell & Developmental Biology (Vol. 20, No. 1, pp. 65-71). Academic Press.

Wittkopp, P.J., True, J.R. and Carroll, S.B., 2002. Reciprocal functions of the *Drosophila* yellow and ebony proteins in the development and evolution of pigment patterns. Development, 129(8), pp.1849-1858.

Sequences:

>HaTH Harmonia axyridis (GenBank accession number: MK584934.1)

GCCCCGAGAGCATCATCTATCATTCTTTGGTTTGCTAAAAACTCGCAAACAGCGTGTTAGAAC
CTGTTGACAAACGGTTATTGATAATCGTGGTCAACTTAACGGTCTCCGAAGACGCTTTCAGTA
GTCAGCAGTGGAAACCATAGTGATTTCAGGCTGAATTTCTCACAGATCCATCTTTTGCGAGTA
GCACCCCCTGTTGAAGTCTCCAGCTCAAAAATGGCCGTAGTCGCAGCAGCTCAGAAGAACAG
AGAGATGTTCGCCATCAAGAAATCTTACAGCATCGAGAACGGTTATCCGGCAAGAAGACGTT
CTTTAGTCGATGATGCAAGATTCGAGACTCTGGTGGTTAAGCAAACCAAACAGACGGTGTTG
GACGAAGCCAGGCTCAGATCTAATGATTCTAGTATGGAGCCTGAAGTTCAACACGAAGACAT
TCCAACCAAAGAAGAAACAACACAAGCGATAAAAGACGATTCTGGACTTACAGAAGAAGAA
GTGTTCCTTCAGAATGCTCTCAGCGAGAATCCTGAATCCGAAAATTACCCGCAAAAGTCCATC
TTGGTCCTGCGTTTGAAAGAAGGAATGAGCTCACTCGGCCGTATCCTCAAGACAGTCGAGAA
GTTCAATGGCTCTGTCTGCCACTTGGAAACCCGCCCTAACAAGAACGAGGGCGCTCAGTTGG
ATGCCCTAATCAAAATCGAGATGTCCAGGATTAACTTGCTCCAAATCTTGAAGTCTCTCCGTC
AGTCTTCTGTTCTGGAGCATTGCACCTTGGTCGGTGACGATAACATCACATCAAAGACACCAT
GGTTTCCAAGACACGCCAGTGAACTGGACAATTGCAACCATCTGATGACCAAGTATGAACCA
GATTTGGATATGAACCATCCAGGATTTGCCGACAAGGAGTACCGTGAACGCAGAAAGGAAAT
TGCTGAGATTGCTTTCTCCTTCAAATACGGTGATCCAATTCCATACATCGAATACGTACCCAC
AGAAATCAAAACTTGGAATTCCGTATTCAACACAGTGCAGGAACTGATGCAAAAACACGCTT
GCGTCGAGTACCGTCGAGTTTTCAAAAAACTGATGGATGAGGGCATCTTTGCTCCTGACAAG
ATTCCACAACTTCAGGAAATGTCCGATTTCATGTGTCGCAACACTGGATTCACCCTTAGACCT
GCTGCTGGACTTCTCACTGCCAGGGATTTCTTGGCTTCTCTCGCTTTCAGAATTTTCCAAAGCA
CACAATACGTCAGACACGTGAACTCACCTTACCACACACCAGAACCAGATTGCATTCACGAA
CTCCTGGGACACATGCCACTCTTGGCTGATCCAAGCTTCGCACAGTTCTCCCAAGAGATCGGT
CTAGCCTCCCTTGGAGCTTCAGATAAAGAAATCGAGAAATTGTCTACGGTATACTGGTTCACA
GTAGAATTCGGCCTTTGCAAGGAGAACGGTAACGTTAAGGCTTACGGTGCCGGCCTCTTGAG
TGCATACGGCGAACTTCTACACGCCCTCAGCGACAAACCAGAACTGAGACCATTTGAACCAG
CAGCCACAGCAGTGCAACCCTACCAGGACCAAGAATACCAACCCATCTACTACGTGGCTGAA
AGCTTCGACGACATGAAAGACAAGTTCAGGCGCTGGGTATCAACAATGTCCAGACCATTTGA
AGTTAGACTGAACGCCTACACAGGAAGAGTGGAAGTCTTGGACTCAGTGGACAAACTTGAGT
CTCTCGTCCACCAGCTCAACACGGAGGTACTTCATTTATCCAACGCTTTGAACAGATTGAAGG
CGCCATCACTTCAGTGAATTGATCTGTACAAAGAAACGGATGCAAGTAGTTAATGTGAGATC
GCATCATCAATGTTATAATCTTTATAATTAGTATCTCTAGAGAATTGAAGACGTGACCCTTTA
TAAAAGTAGCGTCCAACGACGTATCAAGAGATATATTTAGAGAAGAAATAAAATATGAATAT
TATCACATTGTTGTATCCAAATGCACTTGAAACTGTGTTGTTTTCTTCGATGCGAAGAGCAATT
AGCGCAGTATATTATTTATTATGAGGATAGTTTGCACGTTATTGATGTATATAGTGAGAACTA
TATGTTTGTAACTTAATATCTTGCAATGAAACTGCGATTATAATTTATTAGCCTATTTAACCAA
TAAAGTTTATTCTGAAGAAAAAAAAAAAAAAAAAAAAAAAA

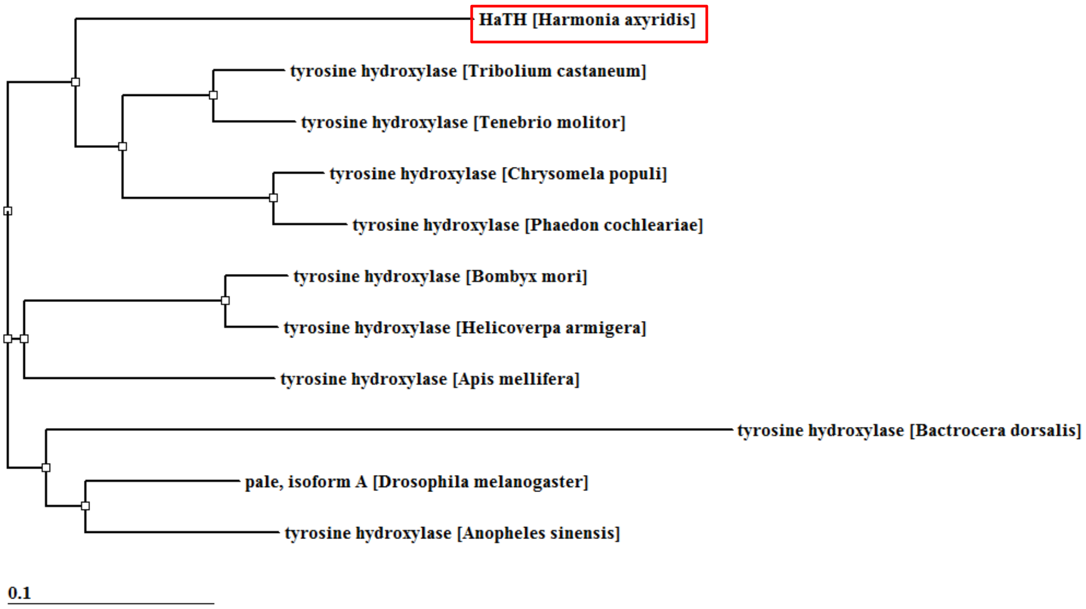

> Halac2 Harmonia axyridis (GenBank accession number: MN650656)

ATGAAGGTGGCGGCAGTACGGTACTGCCTTGTCGCGTTCGCATCAGCGTTGTTCGTCATCCCA
GAAGGCCTTGCCGTGAGAGTTGGCCAGTCAGGAGCACAGAGAAAAGCATCAGTTGCAGACC
AACAGGCTGCCGCATCGTATTGGGATGGAGATTTGGGAAGTGACGTATTTTCTACAGGACAC
ACCCTTCATCAGTCCCATCCACCGGTGGGATCCAGATTCAGGGGCAATGGTGGTAAAAGGAC
AAGTTTAGGAGGACCACCTTTCAAACATTTGGATTATAAAACATCAGCAACAGCAGAATTGA
GAAACAACCCATCTCTATCAGCACCAGAGGAATGTGCTAGGGCTTGTAGAGAAGGAGATCCA
CCAAGGATCTGTTATTATCATTTTACACTTGAATTGTACACCGTACTTGGAGCTGCTTGCCAA
GTTTGTACACCAAATGCCACGAATACAGTGTGGTCCAATTGCCAGTGCGTATTAGCGGATGGT
GTAGAAAGAGGTATATTGACAGCAAATCGTATGGTACCAGGTCCAAGCATACAGGTATGTGA
AGGCGACAAAGTAGTTATCGATGTAGAAAACCACATGGAAGGTATGGAAGTTACCCTCCATT
GGCACGGAATTTGGCAGAGAGGATCTCAATACTATGATGGTGTTCCTTTTGTGACCCAATGTC
CCATCCAGCAAGGAAATACTTTCAGATACCAGTGGGTGGCAGGAAACGCAGGAACCCATTTC
TGGCATGCGCACACAGGTCTACAAAAAATGGACGGTCTTTATGGAAGCATTGTTGTTCGTCAA
CCACCTAACAAAGACCCCAACAGCCACCTTTACGACTATGACTTGACCACCCACGTGATGTTG
CTCAGTGACTGGATGCACGAAGATGCTGCCGAGAGGTTCCCAGGTAGATTGGCTGTGAACAC
TGGTCAAGACCCTGAGAGTTTACTCATCAACGGAAAGGGTCAATTCAGAGATCCAAATACTG
GATTTATGACAAATACTCCATTGGAAGTGTTCACCATGACGCCAGGCAACAAATACAGGTTC
AGGATAATCAATTCCTTTGCTTCCGTTTGTCCCGCTCAACTCACGATTCAAGGACACGATCTT
ATCCTTATTGCTACAGATGGTGAACCTGTGCAACCTGTGACTGTGAACACGATCATCTCATTC
TCTGGTGAAAGATACGATTTCGTCATACACGCCAATAGAAATCCAGGTGCCTACTGGATCCA
ATTGAGAGGTCTAGGAGAATGCGGTGTTCGTAGAGTACAACAACTTGCCATCTTGAGATACG
CTAGAGGTCCATACCAGCCATCTTCACAACCACCAACCTACGATTTCGGTATCCCTCAGGGAG
TGGTTCTTAACCCGTTGGATGCAAGATGTAACGAGCAAAGAGATGACGCAATTTGTGTCAGT
CAATTGAAAAATGCCAAGAGCATTGACCCTGGCATTTTGCAAGAACATCCAGATGTCAAGAT
TTTCTTACCTTTCCGATTCTTGCTCTACACCCCAGAAGAACTTTTCAGGCCAAACACTTACAAC
AGACATTTAGTTGCACCCAATGGTGACCACGTCCTCAGTTTGATTGATGAAATATCATACATG
GCTGCCCCTGCACCACTGATCTCTCAGTACGATGACATAAACCCTGAACAGTTCTGTAACGGA
GACAATAAACCAGCAAACTGTGAGCAAAACTGCATGTGTACTCACAAAGTAGACGTACCACT
GAACGCAATCGTTGAAATTGTGTTAGTTGACGAAGTTCAACAACCCAATCTCAGTCATCCCTT
CCATCTTCACGGTTATTCCTTTAACGTTATCGGTATTGGCCGATCTCCTGACAGGAACGTCAA
GAAAATCAATTTGAAGCACGCCCTTGATCTTGACAGGCAAGGATTGCTTCACAGACAGTTCA
ATCTACCACCAGCTAAAGATACCATCGCTGTGCCAAACAATGGTTACGTTGTGCTCAGACTTA
GGGCAGATAACCCTGGTTTCTGGCTTTTCCACTGTCACTTCTTATTCCATATCGTTATTGGAAT
GAATTTAGTCCTTCAAGTTGGCACACACGCCGATTTACCGCCCGTACCACAAGGTTTCCCAAC
CTGCGGAGATCACTTACCGCCCATATCGTTAGACCCGACAAAAGTTATATAG

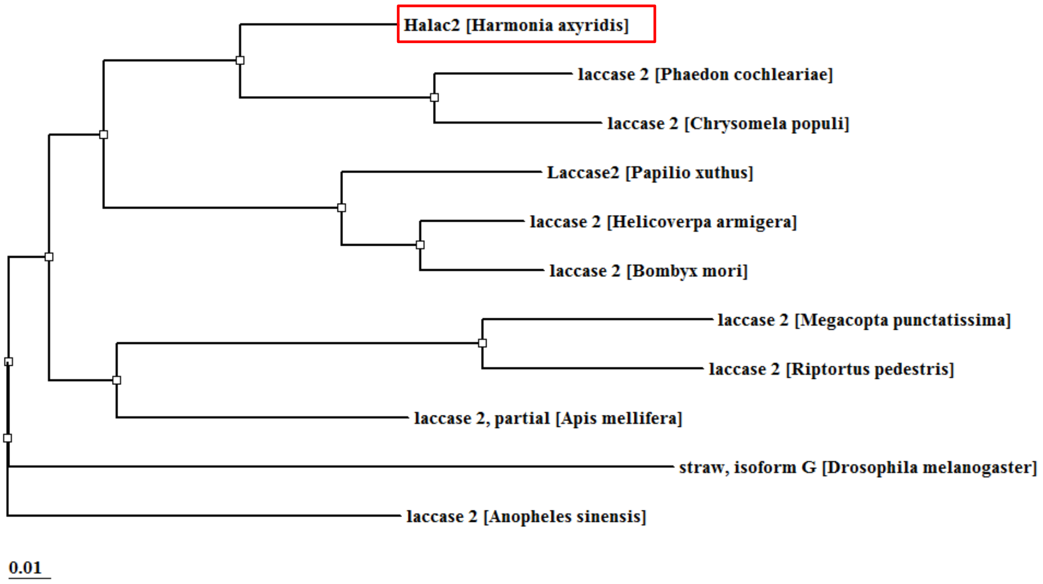

> Haebony Harmonia axyridis (GenBank accession number: MN650657)

ATGGGGTCCCTTCCTCAATTCTCGATTTTGAAAGGACCTACCAGGAAATTCAACCCTGAATTC
ATCAATGACATAATTGAAAGCAGGGTTGGAGATTCGAATACAGCTGATAAAATAGCATTGAT
ATTCGAAGATGGTGATGGAAACACATCAAAACACACCTATGCAGAACTCAACATCATCACCA
ACAAGTTGGCCAGGGTCATCAAGAACACGATCGTACAAGAAAAACTGCAGAGGAACCTTGAT
GGCGACTATTTGGTAGCTGTCAATCTGTTACCAACCGATCGGTTAGTGTTGGTACTACTGGCC
ATTTGGAAGGCAGGAGCTGCATATCTGCCATTGGATCATGCTTTCCCTGGTGCCAGAATTGAA
CACATAATGAGAGAGGCCAAGCCTGCCCTGGTCATTTTTGATGAAGATTCTGATTTCTACGTC
GATGCCTTCAAACTATCTATAGACGAGCTTTGGTCAAAATCTAGCAAAGAATCTGAGATGGG
AATAAAGAAAAACGAACGTCTGAAGCATCAAAGTGGAGACTTGGCTATAGCTCTGTACACAT
CGGGAAGTACAGGAGTACCCAAAGGGGTGAAATTGCCTCACAAGGTGATACTGAATCGTCTG
AATTGGCAGTTCAAGGCCTTCCCTTTCAGTGAAACTGAAAAGGTCTGCGTTTTCAAGACAGCC
TTAACTTTCGTCGACAGTATTTCAGAAATTTGGGGACCTCTAATGAGAGGTCTATCTTTGCTG
GTCATACCCAAAGAGGTCACCAAAGATCCAGAAAGACTCATTGAAGCCTTGGAGAAGTACAA
GGTAGAAAGATTGGTTCTAGTGCCATCCCTGTTGAGATCCATCCTGATGTTCGTTGAAATAAA
GAAACAAGATGGAATCCTGTCCAATCTGAAGACGTGGGTTTGCTCTGGTGAGACCTTGGTCA
AGGGTGCAGCCTTGGACTTCTACAAGTATTTCCCGGAGAACGAACATCGCCTATGCAACTTCT
ATGGCAGTACCGAGATCATGGGTGACGTCACCTATTACGTCATCAAGGGATTGGATCAGATG
AAATCTTTCGAGAAGGTGCCTATAGGTAATCCCGTCGACAACACCATCGTCTATCTCTTGGAC
TCTGAGTTTCGACCAGTAAAAGCGGGAGAGATCGGTGAACTGTACGTTTCAGGCCTCAACCT
GGCCTCCGGATACATAAACGGCAGAGACCCTGATAAATTCATCGAAAACCCCTTGGCTATCG
ATCCTACCTACGGTAAATTGTACAGAACTGGAGATTTTGCACGATTGGAGAAAGGAACCCTG
ATGTACGAGGGTAGGACGGACTCTCAGGTCAAGATAAGAGGTCACCGTGTGGATCTGACGGA
GGTAGAAAAGGCGGTGTCTTCCATTGAAGAAGTGGACAAGGCAGTGGTCCTCTGCTACAAAC
CTGGAGAGATGAGTCAGGCTCTTCTGGCCTTCGTTACCACAAAACAACTGGTGAGCGAGAGT
TGGATCGAGGCATACCTGAGGAAGAAGCTGACGGCCTACATGATACCGCAGGTGATACTGGT
AGAGACTATACCGCTTCTGGTCAATGGAAAGATCGACAGGCAGGGCCTGCTCAAGATGTACG
AGAATACGAACAATAATGACGACTACCAGTATCAAGTGGACATAGACTACACGGGCGTTCCC
TCCAATCAGTTGGAGGCTGCCAAGGTGCTCTTCGAAACAGTGGCGTCCGTCTTGAATAGGGCC
GCCAGGGCTGTCATCAAATCGGACGCAAATTTCTATAATCTGGGTGGAAACTCCCTGAATTCG
ATCTACACTATCACCAAACTGAACGAACAGGGCTACAGAATCGGTATTAGCGATTTCATAGC
AGCTCTGGACCTTGGGGAGATCTTGGAGAGGATGACAGCTGGCACCGTGGTCGATATACGCC
CCCCTCAGTTCACAGCACAGGCCTGCCGTCTCGCCGACAAGGAGATAGTCACTTCGATGATA
ACGGAAAGTTTCTACAGAAAGGCGGATTTGGAACAGTGGATCCTATCGGAAATTTCAGAAAA
CGATTACAGAGTGTACTTGGACGACATGTGGGACCCGTTGGTGGAGAAAGGACTCAGCTTCA
TCGTGAAAAACGAGTATCAGAAAGTTGTTGGCGCCTGCATCAACTTTGATCTCGTGGACGAA
CCTGAAGTGGACATCAGTTCAGGACTGCTTAAGATCTTCGAGTTTCTGGATTTCGTAGAAGGA
CCCTTCAGGCAATCCAAGCTGCCCAAAGAAAAAAACAAAACTCTACACTGTCACATGATGGG

TACCCATAGTTCCTTAACATCGAGGGAAAACATATTGGTCATCCAGTTCATGGAAGAAGAAG
TATACAAACTTGCCAAAAACAAAGGATTCGAAGGAATACTTACCGTTAATACTAGTCCCTTA
ACACAGCAATTGGGCAGAGACGTATTCAAGTACGAAGTTCTGCAAGATTACCAGGTGAATCA
ATATGTAGCTCCAGACAACACAAAACCATTTGGTCTTGCTCCAGACTCCCAAAGGGCTCTGAT
TCAATGGAAAGCTGTAGTTTAA

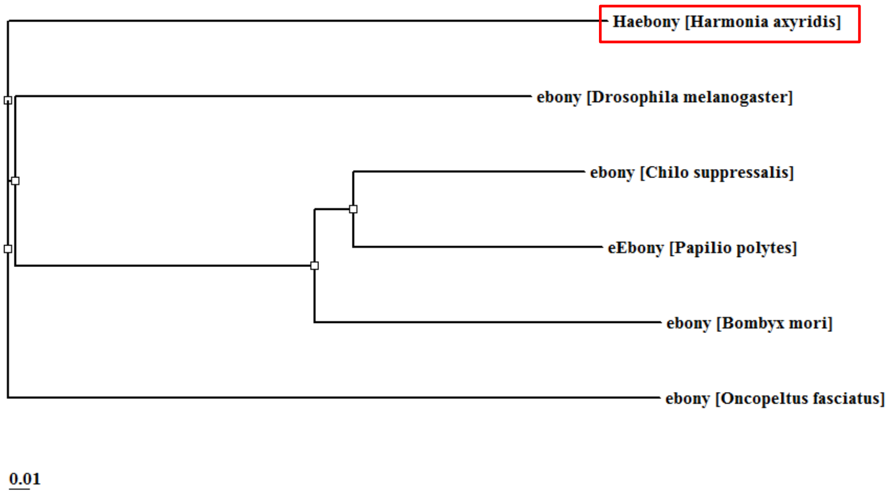

> HaADC Harmonia axyridis (GenBank accession number: MN650660)

ATGCCTGCAGACAACGTGGAGATCGTTTCCTACGACAAATTCTCGTTGTCTTCCTCGTCCTCG
GATGAAGAATACTCGAGTTACAGGAAGGTCGACTCTTCAGACGATGAAGGCATACAGTTCGC
CGTGACTGGTGATCCTAAGGACGAAAGCAACGAGGTCCACTTTGAGAGCTTACCCAGGAAGG
ACCTACACGAGGCTTTCCTGAAAAAATGCGTGGATGTAATATTGAAAGAGGCAGTTTTTGAA
GGCACCAAGAGGCAGAATAAGGTCACCAGCTTCAAGAGCCCTGAGGAGTTGAGAAGTCTCTT
CAACTTTGAGCTTAAAGATGGTGCTTCTAGCCATGATAAGCTCTTAAGAGTGGTTAGGGATAC
GATTGCGTATTCGGTGAAGACTGGACACCCGTATTTTGTGAATCAGCTGTTTTCTTCGTTGGA
CCCTTATGGTCTGGTGGGACAATGGCTTACAGACGCTCTAAATCCTAGCGTTTACACTTTTGA
GGTGTCGCCGGTATTTACTTTGATGGAGGAAGAGGTGTTGAAGGAGATGAGGAGGATTGTTG
GGTTCCAAGGTGGTGAAGGTGATGGTATATTCTGCCCTGGAGGGTCTATGGCTAATGGTTATG
CCATCAGTTGCGCTAGGCATAAATTTTTTCCGGATGCTAAGACCAAAGGCCTTCACTCTCTAC
CGCGCTTAGTTTTGTTCACCTCAGAAGATGCCCACTATTCTATCAAAAAGTTGGCTTCGTTCG
AAGGACTTGGTTCAGACAACGTCTATCTCATCAAGACTGATTCGAGGGGAAAAATGGACGTC
AAGGACCTCGAGAGCCAAATAGAGAGAAGCTTGAAGGAAGGTGCTGCACCATTTATGGTCAG
TGCAACCTCCGGTACAACAGTGTTAGGAGCTTTTGACCCGCTAAACGAAATTGCTGACCTTTG
CCAGAAACACAAACTCTGGCTTCATGTTGACGCTGCTTGGGGTGGCGGGGCTCTGATGTCCCA
GAAGCATAGACATCTCCTAAGTGGCATAGAGAGAGCAGATTCAGTAACATGGAACCCCCATA
AGTTACTTACAGCACCACAACAATGTTCAACTCTCCTACTTAAACACAAAGGACTCCTTGCAC
AAGCACATGGAACTGGAGCTGGTTATCTTTTCCAGAAAGATAAATTTTATGACACTAAATACG
ATACAGGGGACAAACATATCCAGTGTGGAAGAAGAGCTGATGTTCTAAAATTCTGGTTTATG
TGGAAAGCAAAGGGAACAAATGGTCTCGTTCAACATGTCGAAAAAGTTTTCGAGAATGCAGC
CCATTTCATCCAGTTGATCAGAGACCGAGAAGGTTTCGAATTAGTTCATCCTACACCAGAATG
TACTAATATCTGTTTCTGGTACATACCGAAAAGTTTACGAAATGCAAAATCAGAAAAAGATT
ATGGAGAACGTTTACATAAAATTGCCCCAGCTATGAAGGAAAAAATGATGAAAGAGGGCACT
ATGATGGTGACTTACCAGTCTCAAAAAGGTCTACCAAACTTCTTTAGGATAGTTTTCCAAAAC
TCAGCTCTGAATAAATCTGATATGGTTCATTTAGTTGAAGAATTCGAAAGGTTAGGCTCAGAT
TTGTAG

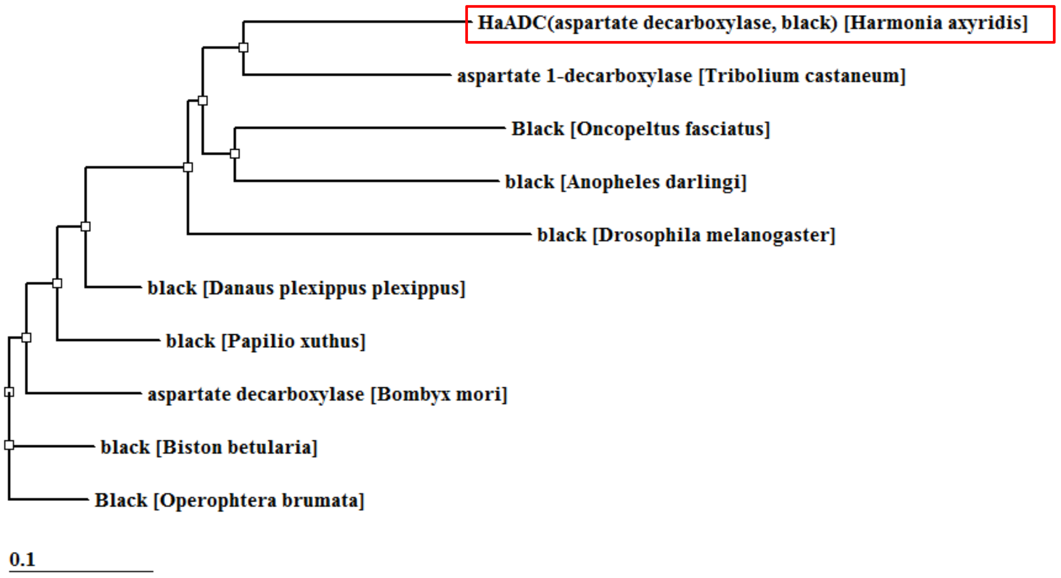

> Hatan Harmonia axyridis (GenBank accession number: MN650658)

ATGCCGGGTACAGGAAATGAACTCGGCAGGCGCCAGAGTATCCCTATTCTTTATACTAGAGG
GACACATTATGAAGTAGGCTTCGATGTGGGAAGGACTTTCGGATCCTTGATTGAAAGTTTCGT
AAATTCATCTACAGCCCTAAATCAAGAATATTTACCTGCTTACAATACACCTGGAGGAAAGC
AAGCCTACGATGCAACTCTCAACAATTTGATTCAGAATTATCCACAATATATTAAAGAATTAG
AAGGAATTGCTGATGGCTCGAAAGTTCCATTCTATAAGCTCTTCTTACTCCATATAGATGACG
TGATACTTCCAACAGTACATAAAGAAGGTTCGAAGCAGCCAACAGGATGTTCTTCCATTGCA
ATCAATTTAAAAGATCAAGCAATTCTTGGACATACAGAAGATGCTCTGAGTGAAACCCTAAA
CCATTTTTATTTTGTGTCTGCTCACATAATCTCAGATAAGCCACAAGGAAGATGGAACGTCAA
AGAAGAAAAATTTACCTCTTTATGCTATGCGGGTCATTTGCCTGGATATACAATGAACTACAA
TCATCATGGTTTAGTTTTTTCGATAAACACAATCAGTGCCAAAGATGTCCAGCCAGGAAAAAC
ACCACGCCATTTTCTGACCAGGGCAATGCTAGGAGCTGAAAATTTTCTGCAGGTCCAGCAGAT
TCTGCGGGATACAGGAGTTGGTGCAGCAGATGCGTGTTCAGTAAACATGACCTTTTTACAGCA
GGAAGGTGATAGAGTGTTCCATAATATCGAAATCGGTCCAGCAATCAATTCTAGTGAAACAC
AACTCAGTGCCTTAACGATAAGTTGTGGAGAACATTTCTACCATACCAATGAATTCCAAAGAC
TGAAAGAAAATCAAGCAAATCCATTAATGCTTCTCAGTAGTAAATGCAGATCTGAAACTTTC
AAAAATCACCATCCGCCTTCTACTTTTGAAGATGTTGTGGAGATGCTGAGTGATACGTCGCAT
CCTGAACAAAAAGTTTTCAGAGACCAACCAAATGATTATGTTAAAACTATTGCAGTTGGAAT
ATTTGACTGCAAGAAGAAGACTTGGAGTCTTTACTCTGATAACCCAAAACACAATGAACCAT
TGGTTGTACTTCCATTAGTTTTGAAGAATTCAAAAACTAATTAA

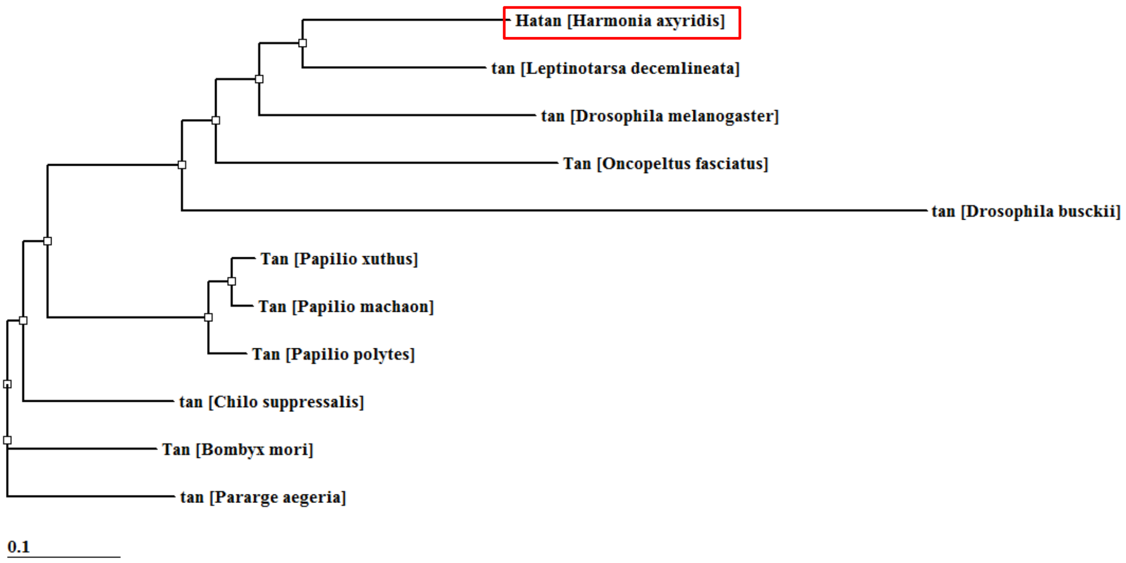

> Hayellow Harmonia axyridis (GenBank accession number: MN650659)

ATGATTTCTATATTACCGAAAGCATCGACATTTCGGGCATTATGGTTCCTTCTAGTCATCTACC
AGGTCCAGGCCACCTACAGGTTACAAGAGAGGTACGCCTGGAACTACATCGATTATGCCTTC
AACGACATCAATCATAAGGTTCAAGCTCAGATAACGCGAAACTACATTCCTGAAAACAATCT
CCCTGTCGGAATTGAAGTATGGAATGACAAAATGTTCATTTCTGTACCAAGATGGAAGGAAG
GTATACCAGCCACACTCAACTACATCTCCCTCACAGACAACAACTCAAAATCCCCTCCTCTAA
TCCCCTACCCCGACTGGAAATCCAACGAGCTAGGAAACTGTAATACCGGCATGAACACAGTC
TACAGAATCAAGGCTGACGAATGCAACAGGCTCTGGGTTCTCGACACCGGAACCTACGGTAT
TGGAAACACCACTCAGCAGCTGTGCCCTTACAGCATCAACGTCTTCAACCTCAGGACCAACC
AGCGGATCAGACGTTTCGAGATCCCAGCTGAGTTGATCAAAGAAGGCACCTTCATCGCAAAC
ACTGTTGTAGATGTGGGAAAGAGTTGCGATGATACTTTCGCATATTTCAGTGACGAATTGGCC
TATGGTATGATTGTGTACTCTTGGGCTGAAAACAGGGCTTGGAGATTCAAGCATGGTTATTTC
TTCCCCGATCCTCTTGCCGGTAACTTCACCATTGATGGGTTGACCTTCAACTGGGATGAGGAA
GGTGTTTTCGGTATGTCCTTGTCACCCCGTTACGTAACCGGTGAAAAGTACCTCTACTTCAGTC
CCTTGGCCAGTAACAGGGAATTTGCAGTATCCACCAACGTCCTCCGTAACAGTTCCAAAGTTG
ACGACAGCTACAATGACTTCATAGCCTTGGAAAACAGGGCACCAAACTCCCACACCACAGCC
AGAGTTATGGACGATTATGGTGTCCAACTATTCAATCTGATCGACCAAAATGCCATAGGTTGT
TGGAACTCTCTTCTTCCTTACCATCCCAAGTACCACTGCGTCGTTGACAGAGATGATGTAGGG
CTTATATTCCCTTCAGATGTCAAAGTTGACGCACATAGAAATGTTTGGGTGATGTCGGATAGG
ATGTCGAACTTCCTCCAGGCTCCAGAAGGCCTTAACTATAACGAGGTCAATTTTAGGGTGTAC
ATGGCACCTATGAAGGTATTGATCCAGGGTACCGTCTGTGATGTAGGTTTGGAAGGCAGTCG
CAGTTACCACGTCAACGAATTGTTCGAATAA

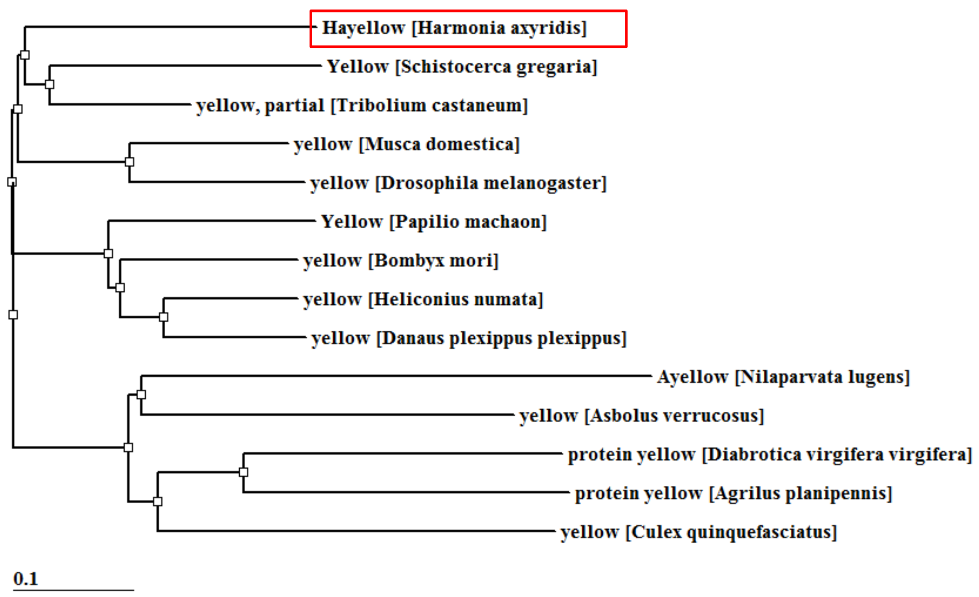
Hayellow [Harmonia axyridis]
Yellow [Schistocerca gregaria]
yellow, partial [Tribolium castaneum]
yellow [Musca domestica]
yellow [Drosophila melanogaster]
Yellow [Papilio machaon]
yellow [Bombyx mori]
yellow [Heliconius numata]
yellow [Danaus plexippus plexippus]
Ayellow [Nilaparvata lugens]
yellow [Asbolus verrucosus]
protein yellow [Diabrotica virgifera virgifera]
protein yellow [Agrilus planipennis]
yellow [Culex quinquefasciatus]
0.1
